# Anion‐Vacancy Activated Vanadium Sulfoselenide With In‐Plane Heterostructure Enabling Durable and Wide‐Temperature Zinc‐Ion Batteries

**DOI:** 10.1002/advs.202502745

**Published:** 2025-03-26

**Authors:** Zhong‐Hui Sun, Wei Zheng, Rui Zheng, Zhen‐Yi Gu, Yu Bao, Zhen‐Bang Liu, Zhong‐Bo Sun, Li Niu, Xing‐Long Wu

**Affiliations:** ^1^ Guangdong Engineering Technology Research Center for Photoelectric Sensing Materials and Devices Guangzhou University Guangzhou 510006 P. R. China; ^2^ MOE Key Laboratory for UV Light‐Emitting Materials and Technology Northeast Normal University Changchun 130024 P. R. China; ^3^ Department of Control Engineering Changchun University of Technology Changchun 130012 P. R. China

**Keywords:** anion‐vacancy, in‐plane heterostructures, self‐powered strain sensors, wide‐temperatures, Zinc‐ion batteries

## Abstract

Zinc‐ion batteries (ZIBs) represent a promising energy‐storage device, which has remarkable merits in terms of cost‐effectiveness, high safety, and environmental sustainability. Transition metal chalcogenides are emerging cathode materials for ZIBs due to their high theoretical capacity and large interlayer spacing. Nevertheless, their application faces critical challenges of sluggish reaction kinetics and huge volume variation. Herein, the anion defect engineering strategy for one‐step in situ anchoring vanadium sulfoselenide on V_2_CT_x_ template (VSSe/V_2_CT_x_) in‐plane heterostructure with built‐in anion vacancy is proposed by robust interfacial C─Se─V bonds to overcome these challenges. The incorporation of the Se atom into VS_2_ not only changes the metal V atom electronic structure and enhances the intrinsic electrical conductivity of VSSe/V_2_CT_x_, but also creates more active sites and accelerates the reaction kinetics as confirmed by theoretical calculations and experimental results. Thus, the VSSe/V_2_CT_x_ cathode delivers a high capacity of 114.3 mAh g^−1^ at 5 A g^−1^ over 15 000 cycles under cryogenic conditions in quasi‐solid state ZIBs (QSSZIBs). Furthermore, the two QSSZIBs successfully integrated with a hydrogel strain sensor enabling reliable human motion and physiological signal detection, highlighting the promise of VSSe/V_2_CT_x_ cathode for self‐powered wearable healthcare monitoring and management systems.

## Graphical abstract

The anion defect engineering strategy for one‐step in situ anchoring vanadium sulfoselenide on V_2_CT_x_ template (VSSe/V_2_CT_x_) in‐plane heterostructure with built‐in anion vacancy is proposed. The incorporation of Se atom into VS_2_ not only regulates the electronic structure of the central metal V atom and enhances the intrinsic electrical conductivity, but also creates more active sites and accelerates the reaction kinetics of VSSe/V_2_CT. As a proof of concept, the QSSZIBs successfully integrated with a hydrogel strain sensor enable reliable human motion detection, highlighting the promise of VSSe/V_2_CT_x_ cathode for wearable healthcare monitoring and management.

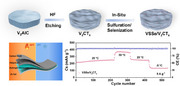

## 1 Introduction

The rise of wearable electronics stimulated the development of high‐safe energy supply devices, making various rechargeable aqueous batteries attract more and more attention. Multivalent metal ion batteries are regarded as promising alternatives due to their high ionic conductivity, environmental friendliness, as well as cost‐effectiveness, which become a research focus in the academic and industrial fields.^[^ ^1^, ^2^, ^3^, ^4^ ^]^ Among them, aqueous zinc‐ion batteries (ZIBs) have been intensively studied due to their high theoretical capacity and good producibility.^[^ ^5^, ^6^, ^7^ ^]^ Though significant progress has been achieved, ZIBs still face challenges, especially for cathodes in terms of high‐rate capability and long durability. To date, various types of cathode materials have been intensively explored including vanadium‐based materials, manganese‐based materials, and organic compounds.^[^ ^8^, ^9^ ^]^ However, most of them suffer from sluggish reaction kinetics resulting in rapid capacity decay under complicated and extreme environments.^[^ ^10^, ^11^, ^12^ ^]^ Therefore, a key solution in achieving fast‐charging ZIBs is to advance the development of cathode materials with rapid reaction kinetics and highly stable reversible capacity.

Layered transition metal dichalcogenides with facile interface editable ability forebode a great potential as Zn^2+^ host materials in ZIBs. Due to the larger atomic radius and stronger shielding effect of Se atom, Vanadium‐based selenide owns the weaker electrostatic interaction with Zn^2+^ along with the weaker metal─Se bond, achieving enhanced electronic conductivity and rapid ion‐diffusion kinetics than vanadium‐based sulfide and oxide.^[^ ^13^, ^14^, ^15^ ^]^ However, the low specific capacity and unsatisfactory rate capability resulting from severe particle agglomeration are urgent to be resolved prior to application. According to the previous reports, creating selenium vacancy at the nanoscale can reduce the interfacial adsorption energy barrier, enhancing the Zn^2+^ storage property of VSe_2‐x_.^[^ ^16^, ^17^ ^]^ In the comparison of layer‐by‐layer self‐assembly ^[^ ^18^ ^]^ and ex situ out‐plane heterojunction configuration^[^ ^19^ ^]^ the in‐plane heterostructure avails face‐to‐face full contact between two active constituents at a mesoscopic scale can further accelerate Zn‐ion transport kinetics and enhance reaction reversibility. Therefore, constructing an in‐plane heterostructure with built‐in anion vacancy at multiscale can maximally unlock its capacity storage limit^[^ ^20^ ^]^ V_2_CT_x_ with abundant redox chemistry of vanadium is selected as a template to study Zn‐ion storage capability by in situ interface phase transition to construct anion‐vacancy enriched in‐plane heterostructures.^[^ ^21^, ^22^ ^]^ The introduction of selenium at the nanoscale and construction of V_2_CT_x_‐based in‐plane heterojunction at mesoscopic can contribute to the prominent surface adsorption, thus boosting the Zn^2+^ reaction kinetics and thermodynamics.

In this work, a self‐confined growth strategy is proposed to prepare VSSe/V_2_CT_x_ in‐plane heterostructure with built‐in anion vacancy through partial substitution of S atoms for Se atom with V_2_CT_x_ as template at multiscale, where the surface V atoms are purposefully transformed into VSSe component through one‐step in situ selenization/sulfuration method, achieving a complete face contact with the inner V_2_CT_x_ layers, buffering the substantial volume strains and guaranteeing robust structural stability during Zn^2+^ insertion/extraction processes. Benefiting from the unique structural design, VSSe/V_2_CT_x_ in‐plane heterojunction shows improved Zn‐ion reaction kinetics, as confirmed by pseudocapacitive analyses, galvanostatic intermittent titration (GITT), and density functional theory (DFT) results. As a result, wide‐temperature, high‐capacity quasi‐solid ZIBs (QSZIBs) can be realized by assembling VSSe/V_2_CT_x_ cathode and PVA‐based hydrogel electrolytes. A high capacity of 114.3 mA h g^−1^ can be obtained after 15 000 cycles under a cryogenic environment, significantly surpassing most of the reported ZIBs performances. Furthermore, a self‐powered sensing device is designed by integrating independent QSZIBs with a PVA‐based strain sensor enabling accurate human movement and physiological signal monitoring.

## 2 Results and Discussion

**Figure 1:**
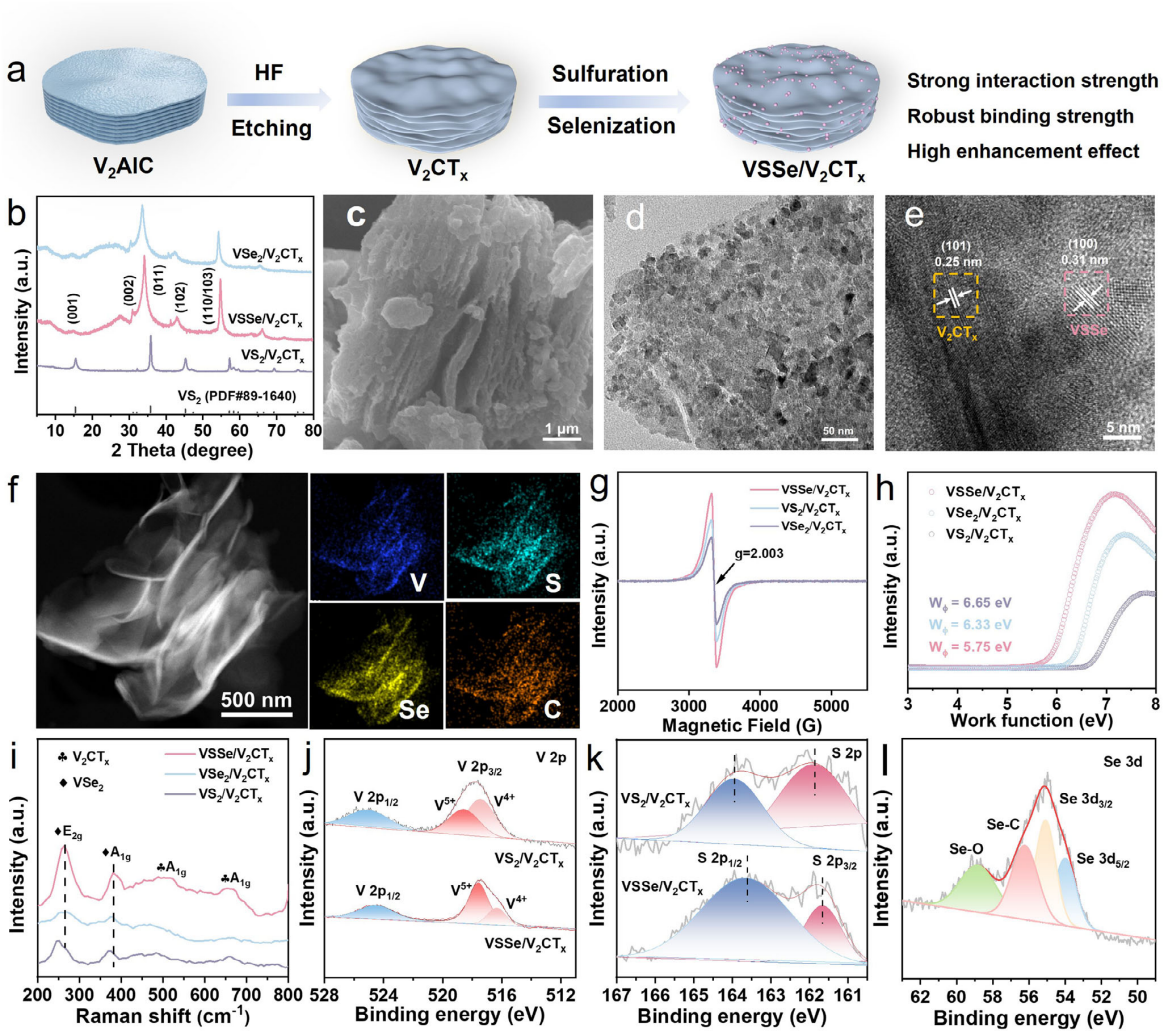
a) Schematic diagram of the synthesis process for VSSe/V_2_CT_x_. b‐l) Structural and morphological characterizations of VSe_2_/V_2_CT_x_, VSSe/V_2_CT_x_, and VS_2_/V_2_CT_x_. b) XRD profile. c) SEM image of VSSe/V_2_CT_x_. d) TEM image of VSSe/V_2_CT_x_. e) HRTEM image of VSSe/V_2_CT_x_. f) EDS elemental mappings of VSSe/V_2_CT_x_. g) EPR spectra. h) UPS diagram. i) Raman spectra. XPS spectra of j) V 2p, k) S 2p, and l) Se 3d.

The synthetic process of VSSe/V_2_CT_x_ in‐plane heterostructure is depicted in **Figure** **1**a. First, V_2_CT_x_ with accordion‐like is obtained by chemically etching the Al layer of V_2_AlC by HF solution (Figure S1, Supporting Information).^[^ ^23^ ^]^ Afterward, VSSe/V_2_CT_x_ nanohybrid is prepared through the synchronous surface selenization and sulfuration reaction under vacuum conditions, during the reaction process, the surficial V atoms are converted into VSSe component, whereas the internal V_2_CT_x_ is deliberately retained, forming VSSe/V_2_CT_x_ in‐plane heterostructure. For comparison, VS_2_/V_2_CT_x_ and VSe_2_/V_2_CT_x_ are also prepared by similar methods, and their morphological characterizations are shown in Figures S2 and S3 (Supporting Information). The crystal structures of the three V‐based chalcogenides are tested by X‐ray diffraction (XRD) technique. As shown in Figure 1b, VS_2_/V_2_CT_x_ shows the typical characteristic peaks of hexagonal phase VS_2_ without any impurity (JCPDS No. 89–1640). It is worth noting that the characteristic peaks of VSSe shift toward lower angles after the introduction of large‐sized Se into VS_2_, which is ascribed to the widened lattice spacing, indicating the mixture of Se and S at the atomic level. The scanning electron microscopy (SEM) image in Figure 1c displays a rough structure with open architectures due to the formation of the VSSe component. The transmission electron microscopy (TEM) image in Figure 1d further confirms that VSSe and V_2_CT_x_ exhibit tight face‐to‐face full interfacial contact constructing in‐plane heterojunction with ultrathin lamellar structure. High‐resolution TEM (HRTEM) image shows clear lattice spacings of 0.31 and 0.25 nm, which are ascribed to the (100) plane of VSSe and (101) of V_2_CT_x_, respectively (Figure 1e). The selected area electron diffraction (SAED) pattern depicts multiple diffraction rings (Figure S4, Supporting Information), indicating the polycrystalline nature of VSSe/V_2_CT_x_. Moreover, the diffraction rings detected in the SAED pattern can correspond to (002), (011), (102), (110), and (023) crystal planes of VSSe/V_2_CT_x_, which is consistent with the XRD result.^[^ ^24^ ^]^ The high‐angle annular dark‐field scanning TEM (HAADF‐STEM) image of VSSe/V_2_CT_x_ and the corresponding elemental distribution mappings display that V, Se, S, and C are uniformly distributed over the VSSe/V_2_CT_x_ (Figure 1f). Electron paramagnetic resonance (EPR) analysis is performed to confirm the existence of anion vacancies. As shown in Figure 1g, compared with VS_2_/V_2_CT_x_ and VSe_2_/V_2_CT_x_, VSSe/V_2_CT_x_ has a sharp EPR signal at *g* = 2.003, which originates from the lattice mismatching degree of Se and S. Ultraviolet photoelectron spectrometer (UPS) shows that the lowest work function of VSSe/V_2_CT_x_ is beneficial for the facile access of electron to sulfoselenide (Figure 1h), thus modulating the electron arrangement. Raman spectroscopy is further performed to confirm the coexistence of VSSe and V_2_CT_x_ in the heterostructures (Figure 1i). The two conspicuous peaks located at 266 and 380 cm^−1^ representing the *E*_2g_ in‐plane and A_1g_ out‐of‐plane vibration modes of VSSe, respectively. Another two broad characteristic peaks centered at ≈520 and 667 cm^−1^ indicate the existence of V_2_CT_x_. N_2_ adsorption–desorption curves exhibit a type‐IV profile featuring a hysteresis loop, indicating the presence of meso/microporous structure in VSSe/V_2_CT_x_. Brunauer‐Emmett‐Teller (BET) surface area of VSSe/V_2_CT_x_ is 49.20 m^2^ g^−1^, which is larger than that of VS_2_/V_2_CT_x_ (25.04 m^2^ g^−1^) and VSe_2_/V_2_CT_x_ (28.06 m^2^ g^−1^) (Figure S5, Supporting Information). Large specific areas and mixed pore structures are conductive to the rapid electron transfer, electrolyte penetration, and expedited hydrated Zn^2+^ transport, thereby significantly improving the zinc storage performance.^[^ ^25^, ^26^ ^]^

X‐ray photoelectron spectroscopy (XPS) is used to investigate the chemical states of the prepared samples. The full survey spectrum in Figure S6 (Supporting Information) confirms the chemical compositions of VSSe/V_2_CT_x_ including the elements V, S, Se, and C. The high‐resolution V 2p spectra of VSSe/V_2_CT_x_ in Figure 1j show that the characteristic peaks at 517.6 and 516.3 eV are ascribed to V^5+^ and V^4+^, respectively. It should be noted that the V 2p peaks move toward lower binding energy for the VSSe/V_2_CT_x_ after Se doping due to the longer bond length of V─Se and the lower electronegativity of Se atoms^[^ ^15^ ^]^ The S 2p high‐resolution spectra also display a similar phenomenon, wherein the S 2p_3/2_ and S 2p_1/2_ peaks in VSSe/V_2_CT_x_ are shifted to lower energies compared to those in VS_2_/V_2_CT_x_ (Figure 1k), confirming the successful Se doping into the VS_2_/V_2_CT_x_ and regulates the electron states around the S species. In the Se 3d high‐resolution spectrum (Figure 1l), the peaks at 53.9 and 55.0 eV correspond to Se 3d_5/2_ and Se 3d_3/2_ respectively, while the peak at 56.3 eV is attributed to the Se─C bond. The peak at 58.9 eV is ascribed to Se─O bond, associated with the formation of SeO_x_ in the air^[^ ^27^ ^]^ The high‐resolution C 1s spectrum, distinct signals at binding energies of 284.7, 285.7 and 288.9 eV reveal the presence of C─C, C─S, and O═C─O, respectively (Figure S7, Supporting Information)^[^ ^28^ ^]^ The above results clearly reveal that the V─Se─C bond ensures the strong interfacial interaction between V_2_CT_x_ substrate and VSSe active component, enhancing the structural integrity, boosting Zn^2+^ diffusion kinetics, and providing high capacity and ultralong cycling stability.

**Figure 2:**
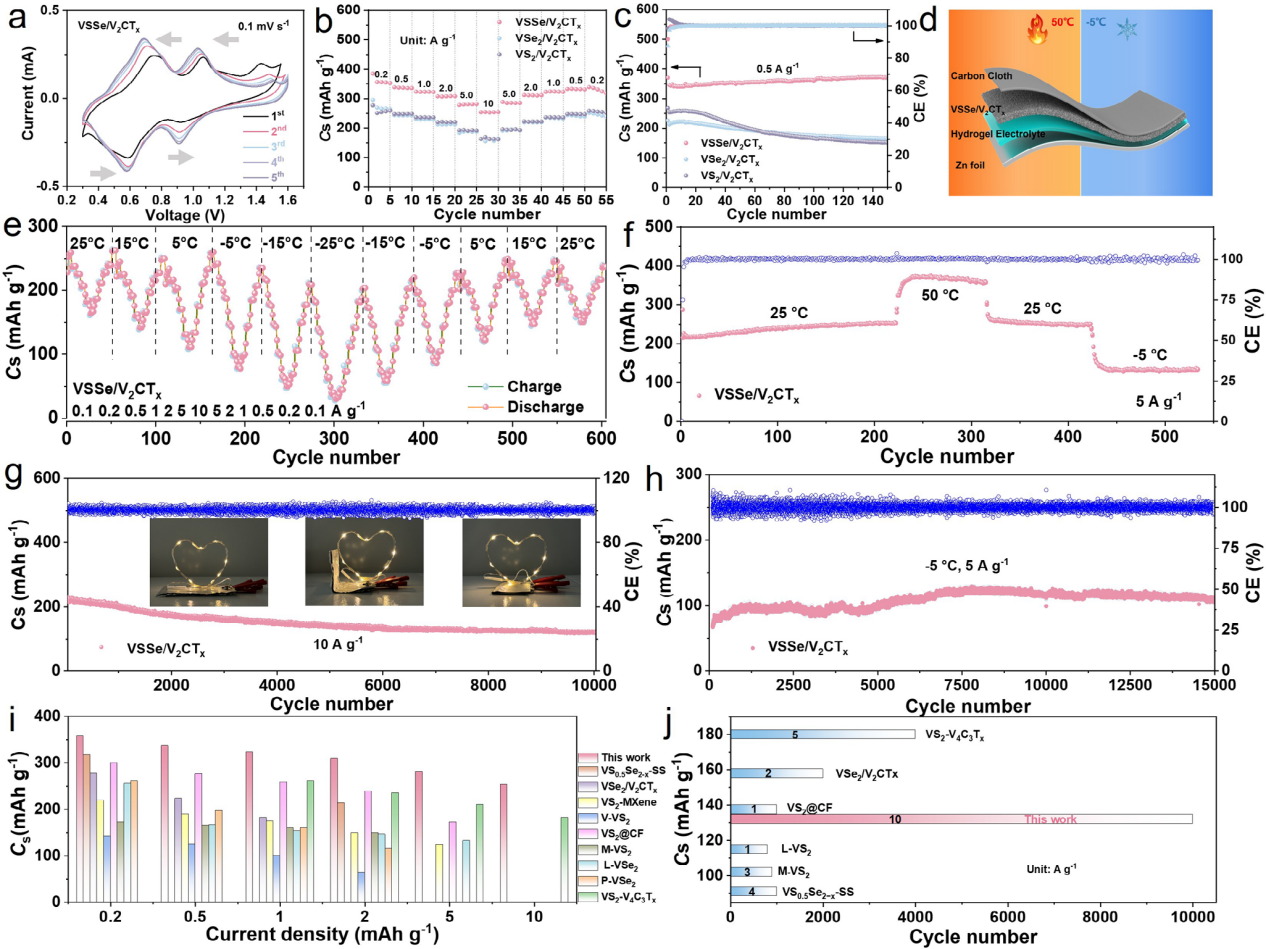
Electrochemical properties of VSSe/V_2_CT_x_ and the two control samples. a) CV curves at 0.1 mV s^−1^. b) Rate capability. c) Cycling stability at 0.5 A g^−1^. d) Schematic diagram of wide‐temperature flexible ZIBs. e) Rate capability at different current densities and temperatures. f) Cycling performance at different temperatures. g) Cycling performance at 10 A g^−1^. Inset: the quasi‐solid‐state ZIB can regularly work under various bending states. h) Long‐term cycling performance at 5 A g^−1^ under sub‐zero condition. Comparison of i) rate capability and j) cycling performance between VSSe/V_2_CT_x_ and other reported vanadium‐based chalcogenides.

The electrochemical performances for Zn^2+^ storage of the as‐prepared composite cathodes are evaluated using half‐coin cells with metallic Zn foil as the counter/reference electrode and 3.0 m Zn(CF_3_SO_3_)_2_ in water as the electrolyte. As shown in **Figure** **2**a, the cyclic voltammetry (CV) curves at 0.1 mV s^−1^ illustrate two pairs of redox peaks ascribing to the stepwise vanadium redox reactions. As the cycles proceed, the two redox peaks' voltage gap significantly decreases, confirming that the reversibility of the electrochemical process is obviously enhanced. It is found that the electrochemical response area of the V^4+^/V^3+^ redox peak is expanded, which resulted from the increased V^4+^ proportion caused by the formation of selenium vacancies. Figure 2b shows the rate capability with different current densities from 0.2 to 10 A g^−1^. The VSSe/V_2_CT_x_ cathode can achieve an admirable discharge capacity of 256.7 mAh g^−1^ with an obvious discharge and charge voltage plateau even at a current density of 10 A g^−1^ (Figure S8, Supporting Information). Moreover, the VSSe/V_2_CT_x_ electrode yields a satisfactory reversible capacity of 371.8 mAh g^−1^ at 0.5 A g^−1^ without any capacity attenuation upon 150 cycles (Figure 2c, Figure S9, Supporting Information). In order to broaden the practical application scenario, VSSe/V_2_CT_x_‐based quasi‐solid state ZIB (QSSZIB) is constructed (Figure 2d). Specifically, the VSSe/V_2_CT_x_ is loaded on carbon cloth as the cathode, zinc plate as the anode, and 3.0 m Zn(CF_3_SO_3_)_2_ filled poly(vinyl alcohol) (PVA) based hydrogel electrolyte as the semi‐solid electrolyte. The structure characterizations of PVA‐based hydrogel electrolytes are shown in Figure S10a,b (Supporting Information), confirming that nano‐SiO_2_ and cellulose nanofibers successfully incorporate into the PVA hydrogel network (denoted as PSC‐hydrogel)^[^ ^29^ ^]^ The mechanical property of PSC‐hydrogel is further studied by the stress‐strain curves, which display excellent robustness under large deformation states (Figure S10c,d, Supporting Information). The improved mechanical properties of PSC hydrogel are mainly due to the reinforcing effects of both cellulose nanofibers and nano SiO_2_ in the PVA networks. Then, the rate capabilities of QSSZIB are first examined at wide‐temperature regions as shown in Figure 2e,f. Unexpectedly, it achieves superior Zn^2+^ storage capability and cycling performance. In this regard, it can deliver a high capacity of 101.2 mAh g^−1^ at 1 A g^−1^ and −25 °C, as well as 370.2 mAh g^−1^ at 5 A g^−1^ and 50 °C, respectively. Moreover, the QSSZIB can maintain a high reversible specific capacity of 120.6 mAh g^−1^ after 10 000 cycles at 10 A g^−1^. It can steadily power LED lights under different bending angles (Figure 2g). Furthermore, ultralong cycling stability can be achieved at 5 A g^−1^ under a sub‐zero environment, with a capacity retention of 114.3 mAh g^−1^ after 15 000 cycles (Figure 2h). Comparative analysis with VSSe/V_2_CT_x_ and other reported vanadium‐based chalcogenides in terms of rate capability and long‐term cyclability are shown in Figure 2i,j.^[^ ^21^, ^24^, ^30^, ^31^, ^32^, ^33^, ^34^, ^35^, ^36^ ^]^ Therefore, it can be concluded that Se doping into VSSe/V_2_CT_x_ crystal lattice in‐plane heterojunction can improve the Zn^2+^ reaction kinetics, thereby achieving excellent electrochemical properties. In addition, the two pouch QSSZIBs connected in series can achieve a high open circuit voltage of 3.05 V (Figure S11, Supporting Information), implying that it can be used for self‐sustaining sensing systems in portable electronics applications.

The above results unequivocally showcase that the distinctive architecture and composition of VSSe/V_2_CT_x_ offer several key benefits for Zn^2+^ storage: Primarily, the existence of in‐plane heterostructure with full facial contact aids in alleviating volumetric expansion during cycling, thereby ensuring exceptional cycle stability. Furthermore, the introduction of larger Se atom doping alters the electronic configuration of the central V atom, expands ion diffusion pathways, and decreases the diffusion energetic barriers, collectively accelerating the redox reaction kinetics. Thirdly, the rich anionic vacancies generate abundant reaction active sites and boost the inherent electrical conductivity, leading to impressive capacitive storage capabilities. As a result, the VSSe/V_2_CTx electrode exhibits superior electrochemical performance for ZIBs.

**Figure 3:**
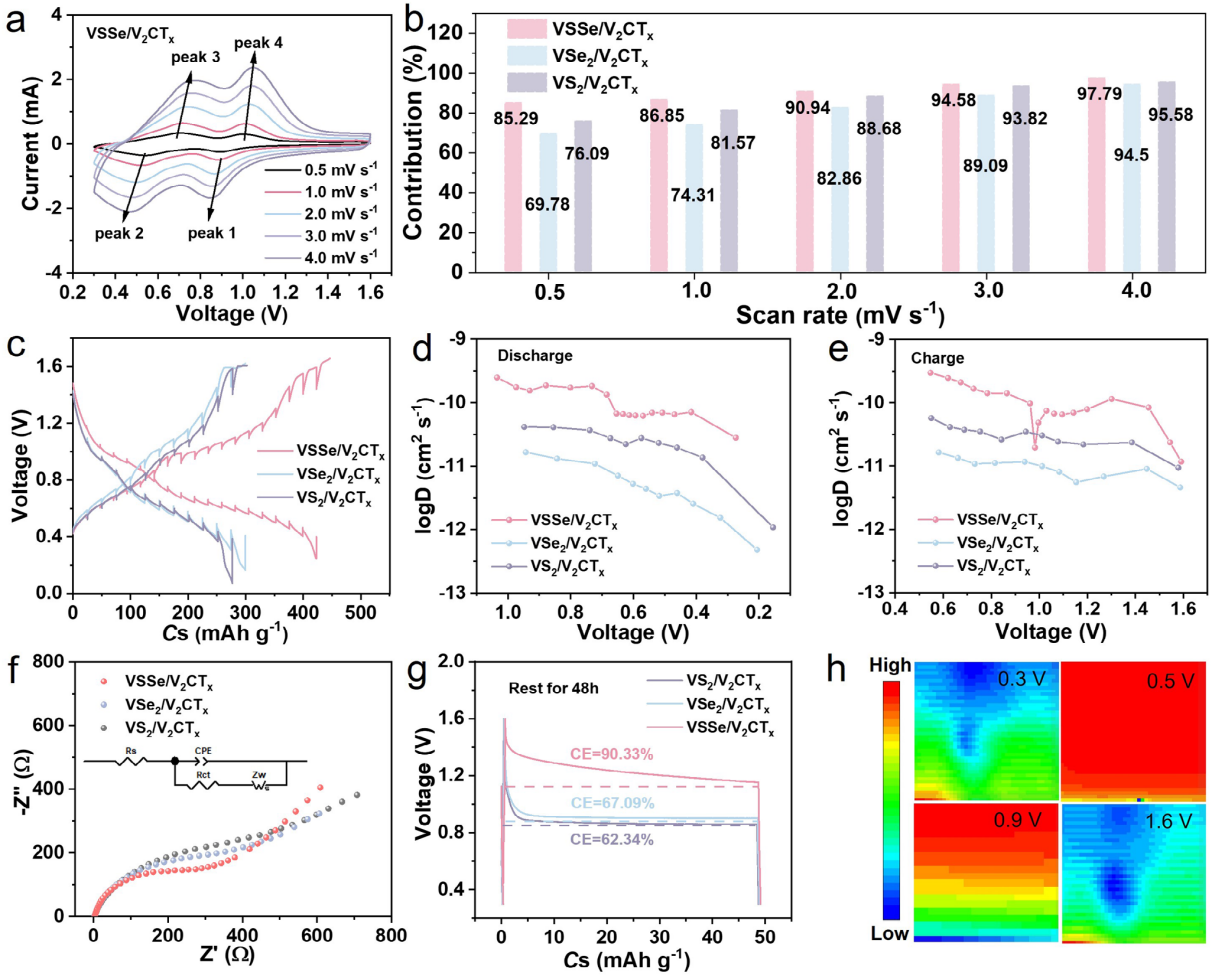
a) CV profiles at gradient sweep rates from 0.5 to 4.0 mV s^−1^. b) Capacitive contribution ratios at various scan rates. c) GITT curves at 0.2 A g^−1^. Zn^2+^ diffusion coefficients during d) discharge and e) charge process. f) Nyquist plots of VSSe/V_2_CT_x_ electrode and its control cathodes. Inset: equivalent circuit diagram. g) Self‐discharge capability curves of ZIBs based on VSSe/V_2_CT_x_ and the control samples. h) SECM area scans of VSSe/V_2_CT_x_ electrode at 0.3, 0.5, 0.9 and 1.6 V.

To further demonstrate the improved reaction kinetics of VSSe/V_2_CT_x_, CV curves are tested with various scan rates from 0.5 to 4.0 mV s^−1^ (**Figure** **3**a). The *b* values of two pairs of reduction peaks are 0.913 and 0.876, respectively (Figure S12, Supporting Information), which are closer to 1, indicating that VSSe/V_2_CT_x_ is dominant by the surface‐controlled process. As shown in Figure S13 (Supporting Information), 97.79% of the total capacity originates from the pseudocapacitive contribution at 4 mV s^−1^. Moreover, with the increase of scanning rate, the proportion of capacitive behaviors gradually enhances, which is much higher than that of control samples (Figure 3b, Figures S14 and S15, Supporting Information). The increased surface‐controlled process indicates the rapid reaction kinetics, consistent with the high‐rate performance of VSSe/V_2_CT_x_ electrode. The galvanostatic intermittent titration technique (GITT) is used to explore the zinc ion diffusion coefficients (D_Zn_^2+^) during the discharge and charge processes (Figure 3c). The calculation results indicate that the VSSe/V_2_CT_x_ electrode has a larger D_Zn_^2+^ compared to VS_2_/V_2_CT_x_ and VSe_2_/V_2_CT_x_ (Figure 3d,e), confirming the enhanced Zn^2+^ diffusion kinetics achieved through the Se doping and tight interfacial integration of the high conductive V_2_CT_x_ substrate. The electrochemical impedance spectra (EIS) are used to explore kinetic properties. As shown in Figure 3f, the three electrodes have similar EIS profiles, including a semicircle in the high‐frequency region and an inclined line in the low‐frequency region. The VSSe/V_2_CT_x_ electrode has a relatively smaller charge transfer resistance and a relatively large slope (Figure S16, Supporting Information), indicating that the introduction of Se effectively improves the charge transfer ability and zinc ions diffusion kinetics, thereby boosting rate capability. The self‐discharge tests in Figure 3g manifest that a slow voltage decay of porous VSSe/V_2_CT_x_ electrode compared to that of VS_2_/V_2_CT_x_ and VSe_2_/V_2_CT_x_ with a higher capacity retention of 90.33% superior to that of VS_2_/V_2_CT_x_ (62.34%) and VSe_2_/V_2_CT_x_ (67.09%) after resting for 48 h. The mitigation of self‐discharge behavior is mainly ascribed to the anion vacancy to strengthen the host structure and inhibit the side reaction. Scanning an electrochemical microscope is performed to investigate the electrochemical reaction intensity of the electrode surface at the microscopic/micron scale. Figure 3h depicts the area scan current distribution and the average current of the modified electrode surface under different polarization voltages ranging from 0.3 to 0.5, 0.9, and 1.6 V, respectively. It can be clearly found that the electrode surface shows significantly larger interface response current and reaction intensity at the potentials of 0.5 and 0.9 V than that at 0.3 and 1.6 V, which can be ascribed to the interactions of anion defect adsorption between Zn^2+^ and VSSe/V_2_CT_x_ in‐plane heterojunction.

**Figure 4:**
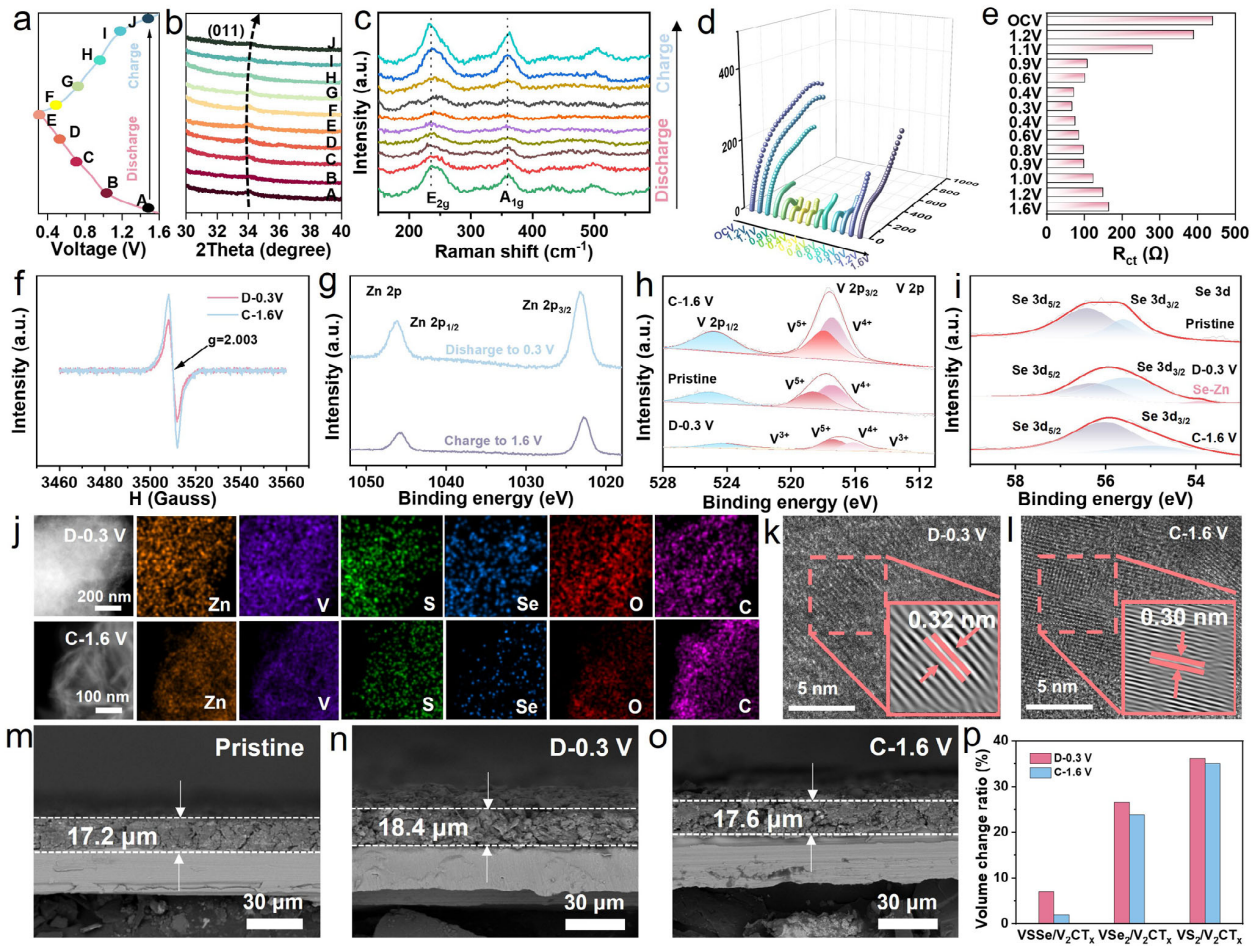
a) Charge–discharge profiles at 0.1 A g^−1^ of VSSe/V_2_CT_x_ in the first cycle. b) Corresponding ex situ XRD patterns. c) Ex situ Raman spectra during discharge and charge process. d) In situ EIS spectra with discharge and charge process. e) Corresponding R_ct_ values variations. f) Ex situ EPR curves of the VSSe/V_2_CT_x_ electrode at different states. g) Zn 2p, h) V 2p, i) Se 3d XPS spectra of VSSe/V_2_CT_x_ at selected voltage states. j) STEM elemental mapping images of the fully discharged and charged VSSe/V_2_CT_x_ electrodes. HRTEM images of VSSe/V_2_CT_x_ at k) fully discharge and l) fully charge states. m–o) Cross–sectional SEM images of VSSe/V_2_CT_x_ electrode at pristine, full discharge and charge states at 1 A g^−1^ after 100 cycles. p) Volume change ratio of VSSe/V_2_CT_x_, VSe_2_/V_2_CT_x_, and VS_2_/V_2_CT_x_ electrodes.

In order to reveal the Zn^2+^ storage mechanism of VSSe/V_2_CT_x_ electrode, ex situ XRD measurements at different voltage states in the 1^st^ cycle are carried out to investigate the structural evolution processes (**Figure** **4**a,b). The (011) characteristic peak shifts from 34.0° to 33.4° in the fully discharged process (from A to E), and then returns to the 34.1° with the charging process (from F to J), confirming the reversible Zn^2+^ insertion/extraction behaviors. Ex situ Raman spectra show the intensity of *E*_2g_ and *A*_1g_ increase first and then decrease with discharge and charge processes, further indicating the reversible Zn^2+^ insertion/extraction process in the VSSe/V_2_CT_x_ (Figure 4c). To illustrate the effect of Se doping on charge transfer kinetics, in situ EIS is performed on VSSe/V_2_CT_x_ (Figure 4d). During the discharge process, the fitted charge transfer resistance (*R*_ct_) gradually decreases because the enlarged interlayer spacing effectively accelerates the Zn^2+^ diffusion (Figure 4e). While in the opposite process, it tends to increase due to the reversible Zn^2+^ extraction^[^ ^37^ ^]^ These results imply that the incorporation of Se effectively increases the electrical conductivity and reduces the interfacial resistance of the VSSe/V_2_CT_x_, thereby improving battery performance. Ex situ electron paramagnetic resonance (EPR) is used to explore the influence of Se defect structure on the zinc storage performance. Figure 4f displays a stronger EPR signal at the fully charged state (1.6 V) than that at the fully discharged state (0.3 V), which may be ascribed to the Zn^2+^ insertion into anion‐vacancy generated additional active sites.^[^ ^38^, ^39^ ^]^ Ex situ XPS analysis is performed to study the change of the main valence state of Zn, V, and Se elements during the charge and discharge processes. For Zn 2p spectra (Figure 4g), the stronger Zn 2p_1/2_ (1046.2 eV) and Zn 2p_3/2_ (1023.2 eV) peaks in the fully discharged states confirm the partial reversible Zn^2+^ insertion. The residual trace Zn^2+^ in the VSSe/V_2_CT_x_ interlayers can serve as “pillars” to effectively reinforce the layered structure of VSSe/V_2_CT_x_ during cycling.^[^ ^40^ ^]^ The V 2p XPS spectra in Figure 4h show that the reversible valence changes between V^4+^/V^5+^ and V^3+^/V^4+^/V^5+^, indicating that V is partially reduced during the discharge process.^[^ ^41^ ^]^ For Se 3d spectra, the Se peak intensity decreases and a new peak at 53.2 eV appears when discharged to 0.3 V (Figure 4i), which might form the Se‐Zn bond due to the Zn^2+^ adsorption by Se vacancies^[^ ^42^ ^]^ STEM‐EDS elemental mapping images manifest the Zn^2+^ insertion/extraction behaviors during the charge and discharge processes (Figure 4j). HRTEM images present that the interlayer distances of the fully discharged and charged electrode materials are 0.32 and 0.30 nm, respectively (Figure 4k), which is in accordance with the ex situ XRD result. The cross‐sectional SEM images and corresponding volume change ratio of the three electrodes at various discharge and charge states are displayed in Figure 4m–p and Figure S17, Supporting Information. The results demonstrate that the relatively smaller volume expansion ratio for VSSe/V_2_CT_x_ is observed after 100 cycles compared to VS_2_/V_2_CT_x_ and VSe_2_/V_2_CT_x_, indicating that the Se doping in transition metal sulfide coupled with in‐plane heterostructure plays a significant role in structural stability to buffer volumetric strain effect, thus enhancing the kinetic behavior of the whole electrode.

**Figure 5:**
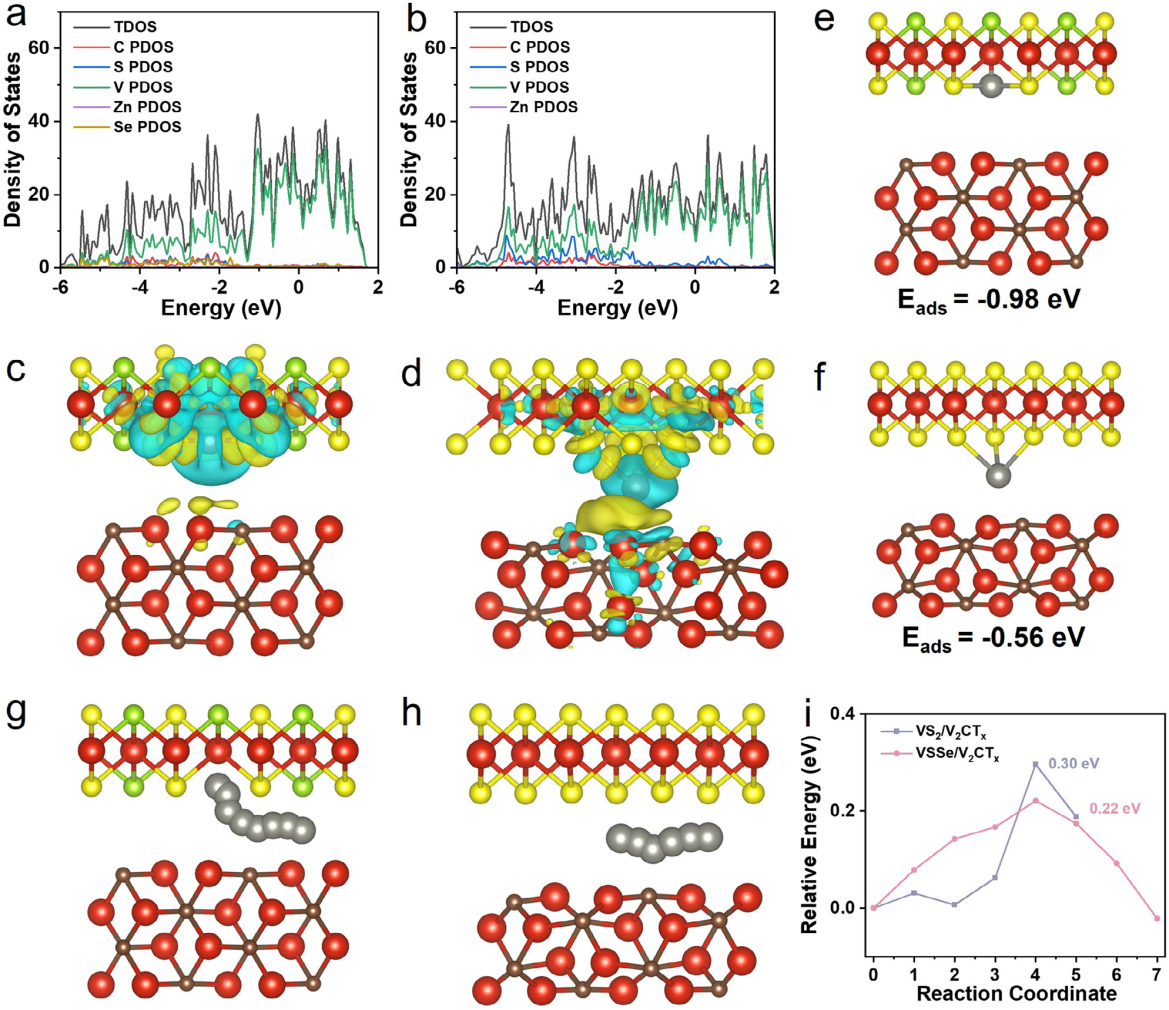
Theoretical investigation of the interaction between Zn atom and VSSe/V_2_CT_x_ and VS_2_/V_2_CT_x_. The TDOS comparison of a) VSSe/V_2_CT_x_ and b) VS_2_/V_2_CT_x_. Charge density difference of c) Zn‐VSSe/V_2_CT_x_ and d) VS_2_/V_2_CT_x_. e,f) Adsorption models and the corresponding adsorption energy. Zn atom diffusion path and energy barrier in g) VSSe/V_2_CT_x_, h) VS_2_/V_2_CT_x,_ and i) their corresponding migration barrier.

Density‐functional theory (DFT) calculations are performed to investigate the fundamental effect of Se vacancies enriched in‐plane heterostructure in VSSe/V_2_CT_x_ electrode on Zn^2+^ storage capability at the atomic level. Two optimized structural models are constructed including VS_2_/V_2_CT_x_ and VSSe/V_2_CT (Figure S18, Supporting Information). The density of states (DOS) is calculated to study the variation in the electronic structure of Se incorporation in VS_2_/V_2_CT_x_ (**Figure** **5**a,b). It is obvious that both VSSe/V_2_CT_x_ and VS_2_/V_2_CT_x_ display typical conductor characteristics near the Fermi energy level, implying their metallic nature and high electronic conductivity. Then, differential charge density distribution is analyzed to reveal the effect of Se‐vacancy with in‐plane heterointerface engineering on the interaction between adsorbed Zn atom and host configuration. It is notable that the yellow and celeste regions represent the charge accumulation and consumption, respectively. As shown in Figure 5c,d, the abundant electron depletion of the Zn atom and accumulation around the VSSe/V_2_CT_x_ can be clearly observed in comparison to VS_2_/V_2_CT_x_, indicating a strong electrostatic interaction between Zn and VSSe/V_2_CT_x_. Moreover, the Zn atom adsorption process for VSSe/V_2_CT_x_ and VS_2_/V_2_CT_x_ is calculated (Figure 5e,f). The adsorption energy of VSSe/V_2_CT_x_ (−0.98 eV) is lower than that of VS_2_/V_2_CT_x_ (−0.56 eV). Furthermore, the Zn^2+^ diffusion paths and diffusion energy barrier of VSSe/V_2_CT_x_ and VS_2_/V_2_CT_x_ are investigated as shown in Figure 5g,h. In detail, the Zn atom undergoes random diffusion across the interlayer of VS_2_/V_2_CT_x_, encountering a relatively high energy barrier of 0.30 eV. However, in the vicinity of the Se‐vacancy (with an energy barrier of 0.22 eV), preferential migration is observed through VSSe/V_2_CT_x_, where the energy barrier is considerably lowered (Figure 5i). Therefore, the coupling effect of the anion Se defect and the interface engineering of V_2_CT_x_ enhances the reaction kinetics by delocalizing the interfacial charge distribution and facilitating additional pathways for Zn atom diffusion. Consequently, this VSSe/V_2_CT_x_ in‐plane heterostructure with anion vacancy exhibits superior structural stability and accelerated reaction kinetics, ultimately enabling highly reversible Zn^2+^ storage capabilities.

**Figure 6:**
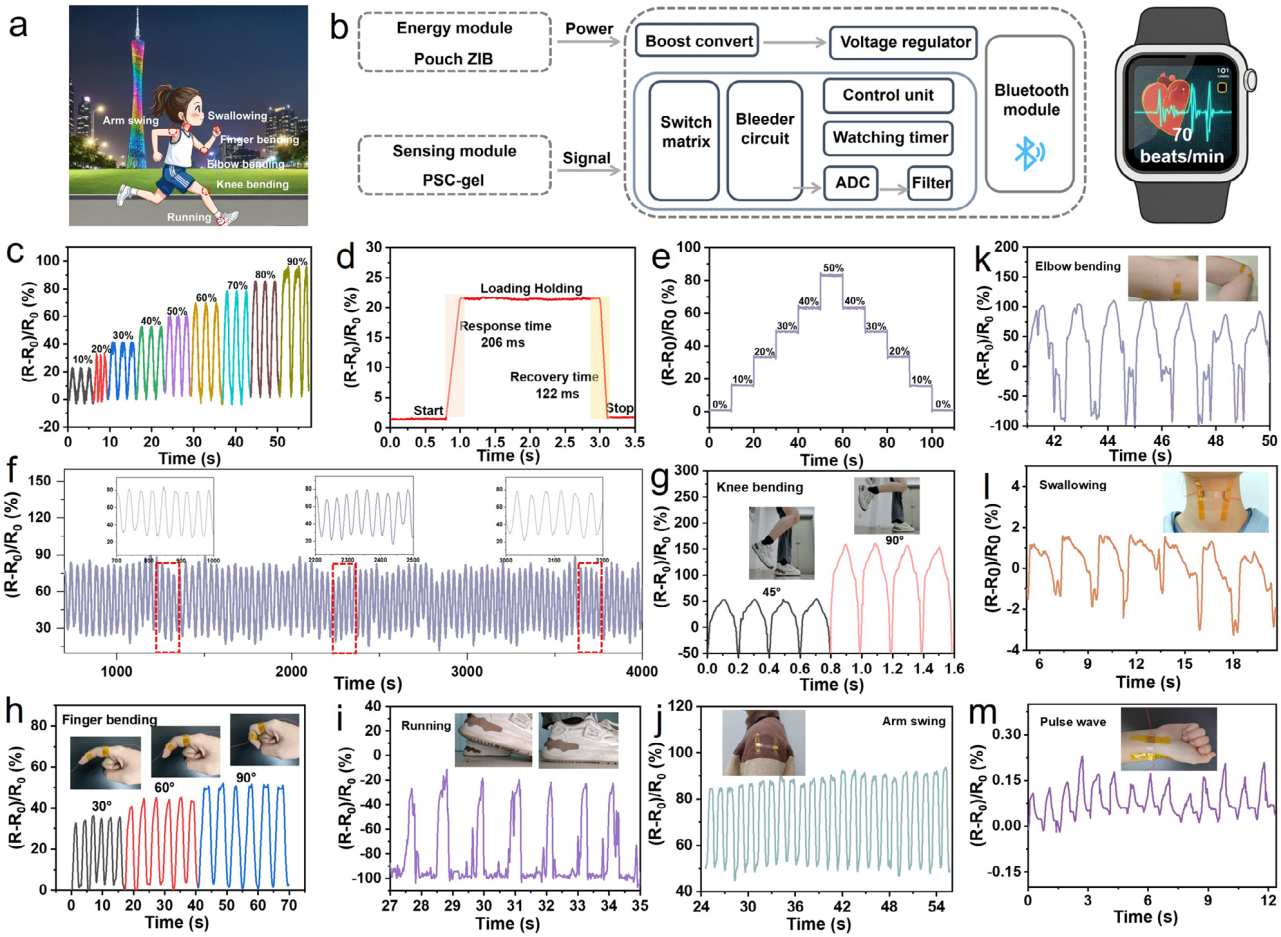
a) Scheme of the PSC‐gel‐based QSSZIBs for real‐time human movement and physiological signal detection. b) Systems‐level block diagram of the self‐powered wearable sensing system. c) Relative resistance changes with the increase of tensile strain. d) Response and recovery time. e) Δ*R*/*R*_0_ values with stepped strains. f) Cyclic tensile loading‐unloading of the flexible sensor. Signals of relative resistance change during g) knee bending, h) finger bending, i) running, j) arm swing, k) elbow bending, l) swallowing, and m) pulse wave.

Encouraged by the intrinsic safety and extended cycling durability of ZIBs, the QSSZIB emerges as a power source for wearable electronic devices.^[^ ^43^ ^]^ Thus, an advanced all‐in‐one wearable self‐powered sensing system is proposed, which integrates an energy supply module, a control module, and a sensing module (**Figure** **6**a,b).^[^ ^44^, ^45^ ^]^ Printed circuit board (PCB) is elaborately designed, including a power management component, a signal conditioning component, and a data transmission component (Figures S19–S21, Supporting Information).^[^ ^46^ ^]^ As a proof of concept, the strain sensor is used in wearable self‐feeding systems to achieve real‐time human motion and physiological signal monitoring. Specifically, the PSC hydrogel synchronously serves as a quasi‐solid electrolyte and strain sensor‐sensitive material, combining the merits of sufficient energy supply, wearable availability, and sensing capabilities (Figure S22, Supporting Information). The Δ*R*/*R*_0_ values of the strain sensor at different stretching strains exhibit a steady signal under the strain range of from 10 to 90% (Figure 6c and Movie S1, Supporting Information), which can completely satisfy the workable range of the sensor during human motion. Sensitivity is a crucial indicator in the evaluation of the sensing performance. Then, the gauge factor (GF) of the strain sensor shows a good linear response in the strain range of 10–90% with a GF of 0.92 (Figure S23, Supporting Information), demonstrating that the PSC‐gel has excellent sensitivity as a strain sensor. Furthermore, the hydrogel displays rapid response time (206 ms) and recovery time (122 ms) when the sensor is under loading and unloading, demonstrating the outstanding electromechanical performance of the strain sensor (Figure 6d). The relative resistance change with a stepped tensile strain under an interval time of 10 s shows the almost complete recovery ability without hysteresis (Figure 6e). Moreover, the durability and stability of the sensor are tested by cyclic stretching‐releasing up to 50% strain, displaying high stability over 1000 cycles (Figure 6f). Based on the superior sensitivity and stability, the strain sensor is mounted at the various parts of the human body for practical movement detection (knee bending, finger bending, running, arm swing, elbow bending). As shown in Figure 6g–k, the Δ*R*/*R*_0_ value regularly responds along with the human joint motions, demonstrating that the strain sensor is suitable for detecting body movement during exercise. In addition to these large motions by human body parts, the sensors also can be used to detect swallowing at the throat and pulse rates at the wrist (Figure 6l,m), demonstrating the high sensitivity of the strain sensor to detect the subtle signals of the human body. The above results collectively confirm the great potential of the self‐powered strain sensing system in the healthcare field.

## 3 Conclusions

In summary, we demonstrate a high‐efficiency strategy to prepare ternary VSSe/V_2_CT_x_ superstructure in‐plane heterostructure with built‐in anion‐vacancy at multiple scales, achieved by in situ synchronous selenization/sulfuration on the surface of V_2_CT_x_. The experimental investigation and DFT calculation demonstrate that this superstructure provides fast redox kinetics and buffers severe volume variations, thereby achieving remarkable zinc storage capability during cycling. As a proof of concept, the QSSZIBs for powering wearable electronics with a strain sensor are proposed. The vertical arrangement of components within the device ensures the efficient integration of energy storage and sensing performances. The battery module demonstrates a specific capacity of 114.3 mAh g^−1^ at 5 A g^−1^ over 15 000 cycles, achieving a fast charge and stable energy supply under cryogenic conditions. The strain sensor module, boasts a high sensitivity, rapid responsiveness, and recovery to external stimuli, accurate real‐time monitoring of human motion, and subtle pulse wave signal. These results highlight the potential application of this device in next‐generation wearable electronics for personalized healthcare.

## Conflict of Interest

The authors declare no conflict of interest.

## Supporting information



Supporting Information

Supplemental Movie 1

## Data Availability

The data that support the findings of this study are available from the corresponding author upon reasonable request.
