# Supplementary material for: Anion‐Vacancy Activated Vanadium Sulfoselenide With In‐Plane Heterostructure Enabling Durable and Wide‐Temperature Zinc‐Ion Batteries
Source: Adv Sci (Weinh). 2025 Mar 26;12(26):2502745. doi: 10.1002/advs.202502745 (PMC12245029; doi:10.1002/advs.202502745)
Supplement: Supplementary file 1 — Supporting Information [file ADVS-12-2502745-s001.docx]

**Supporting Information**

**Anion-Vacancy Activated Vanadium Sulfoselenide with In-Plane Heterostructure Enabling Durable and Wide-Temperature Zinc-Ion Batteries**

*Zhong-Hui Sun, Wei Zheng, Rui Zheng, Zhen-Yi Gu, Yu Bao*, Zhen-Bang Liu, Zhong-Bo Sun, Li Niu*, and Xing-Long Wu**

Dr. Z.-H. Sun, Miss. W. Zheng, Mr. R. Zheng, Prof. Y. Bao, Dr. Z.-B. Liu

Guangdong Engineering Technology Research Center for Photoelectric Sensing Materials and Devices, Guangzhou University, Guangzhou 510006, P. R. China, E-mail: niuli@mail.sysu.edu.cn, baoyu@gzhu.edu.cn

Dr. Z.-Y. Gu, Prof. X.-L. Wu

MOE Key Laboratory for UV Light-Emitting Materials and Technology, Northeast Normal University, Changchun 130024, P. R. China, E-mail: xinglong@nenu.edu.cn

Prof. Z.-B. Sun

Department of Control Engineering, Changchun University of Technology, Changchun 130012, P. R. China

**Experimental Section**

**Preparation of** **V_2_CT_x_**. 1 g of V_2_AlC (300 mesh) was slowly added to 20 mL of 40% HF solution with stirring, after which the mixture was stirred for 120 h at 35 °C. The dispersion was then centrifuged at 5000 rpm for 5 min to harvest the sediment, which was further centrifuged and washed with deionized water until the pH value approached neutral. The sediments were vacuum dried at 80 °C overnight to provide V_2_CT_x_ for further characterization and experiments.

**Preparation of VSSe/V_2_CT_x_, VSe_2_/V_2_CT_x_ and VS_2_/V_2_CT_x_.** 0.2 g of V_2_CT_x_, 0.4 g of Se powder, 0.4 g of S powder (99.99% Aldrich) were mixed and ground for 30 min. The mixture was sealed in quartz tube and then annealed at 550 °C for 2 h with a ramp rate of 2 °C min^-1^ under vacuum. After cooling to ambient temperature, VSSe/V_2_CT_x_ nanohybrids were obtained. For comparison, VSe_2_/V_2_CT_x_ and VS_2_/V_2_CT_x_ were prepared by similar method.

**Preparation of PSC Hydrogel.** A CNF aqueous suspension (10 g) with nano-SiO_2_ (250 mg) was added to EG (10 g) and stirred for 2 h. Then, PVA (5 g) with 3.0 M Zn(CF_3_SO_3_)_2_ aqueous solution (20 mL) was added to the suspension and heated to 90 °C with continuous stirring until PVA was fully dissolved. The PSC mixture was settled in the oil bath without stirring to remove air bubbles, and then it was transferred to a PTFE mold and placed in refrigerator for 12 h for polymerization. After the reaction, the hydrogel was treated with several freezing/thawing cycles (-20 °C for 3 h and room temperature for 3 h) to form PSC hydrogel.

**Assembly of integrated wearable sensing system.** The integrated wearable sensing system was comprised of an energy module, sensing module, controlling module, data transmission module, and display module. The sensor of hydrogel was integrated into a printed circuit board (PCB). The microcontroller unit (STC32G12K128) and Bluetooth module were welded on the PCB and responsible for real-time processing and data transmission, respectively.

To fabricate strain sensors from hydrogels, PSC hydrogel is tailored into strips (50 mm×10 mm×1 mm) with two outstretching conductive tapes. The two ends of the sensor strips were connected to the PCB to measure the resistance variation. The real-time responsive resistance variation is collected by the software of the Bluetooth serial port on a smartphone.

**Material Characterizations**

The morphologies and microstructures were carried out by scanning electron microscopy (SEM, JSM-6700F), transmission electron microscopy (TEM, JEM-2100F) and high-resolution transmission electron microscopy (HRTEM, Tecnai G2). X-ray diffraction (XRD) patterns were recorded on an X-ray diffractometer (Rigaku-Ultima IV with Cu Kα radiation) using Cu Kα radiation (λ=0.15406 nm). X-ray photoelectron spectra were obtained on a Thermo Electron Corporation ESCALAB 250 XPS spectrometer. Raman spectra were performed by HORIBA. Fourier Transform Infrared Spectroscopy (FTIR) spectra were measured by Nicolet 6700.

**Electrochemical measurements**

The cathode slurry was prepared by mixing active materials, acetylene black (CB) and polyvinylidene fluoride (PVDF) with a weight ratio of 70:20:10 in N-methylpyrrolidone (NMP) solvent and then was coated on commercial carbon cloth followed by drying at 80 °C for 12 h in a vacuum oven. The mass loadings of the active materials were about 3 mg cm^-2^. Zinc foil was used as both counter and reference electrodes, and glass microfiber filter (Whatman) as the separator. CR-2032 coin cells were assembled in air using 3.0 M Zn(CF_3_SO_3_)_2_ aqueous solution. Pouch cell was assembled by using PSC Hydrogel as quasi-solid electrolyte. Galvanostatic charge/discharge tests were performed on Land battery test system. The voltage windows were 0.3-1.6 V vs. Zn^2+^/Zn. Electrochemical impedance spectroscopy (EIS) was conducted on an electrochemical workstation (CHI-760e) from 10^5^ to 10^-1^ Hz. Cyclic voltammetry (CV) profiles were recorded on an electrochemical workstation (CHI-760e). GITT tests were conducted by discharging and charging the cells at 20 mA g^-1^ for 30 min with a rest interval of 2 h in the range of 0.3 to 1.6 V.

**Ex-situ X-ray diffraction measurement.** Ex-situ XRD patterns of VSSe/V_2_CT_x_ electrode were collected on the X-ray diffractometer (Rigaku-Ultima IV) for the first cycle in the 2θ range from 20 to 60° with a scanning rate of 5° min^-1^. A current density of 20 mA g^-1^ is selected for charging and discharging processes.

**The calculation process of the capacitance effect and pseudocapacitive contribution:**

The capacitance effect can be determined from the curve, according to the relationship between measured peak currents (*i*) and scanning rates (*v*), as follows:

*i*= *a v^b^* (1)

log *i*= *b* log *v* + log *a* (2)

where a and b are the fitting parameters, and *i* and *v* represent peak current and scanning rate, respectively. For diffusion-controlled behavior, the b-value approaches 0.5, while for a surface capacitance-dominated process, it is close to 1.0.

Moreover, the pseudocapacitive contribution can also be calculated by the following equation:

*i*= *k*_1_*v* + *k_2_v*^1/2^ (3)

where *k*_1_*v* and *k*_2_*v*^1/2^ represent the pseudocapacitive contribution and the ionic diffusion contribution, respectively.

**The details of the diffusion coefficient:**

For galvanostatic intermittent titration technique (GITT) analyses, the cells were cycled in the potential window of 0.3-1.6 V vs. Zn^2+^/Zn at 20 mA g^-1^. The pulse time for each applied galvanostatic current and rest was 1 hour and 6 hours, respectively.

The diffusion coefficient can be worked out by solving Fick’s second law according to Equation

$D=\frac{4}{\pi\tau}\left( \frac{m_{B}V_{M}}{M_{B}S} \right)^{2}\left( \frac{\Delta ES}{\Delta E_{\tau}} \right)^{2}$ (4)

where *τ* is the duration of the current impulse (s), and *m_B_*, *V_M_*, *M_B_*, and *S* are the mass, the molar volume of the active material, the molar mass, and the area of the electrode, respectively. *ΔE_S_* represents the quasi-thermodynamic equilibrium potential difference between the potentials before and after the current pulse. *ΔE_τ_* is the voltage difference during the current pulse.

**Theoretical calculation**

Density functional theory (DFT) calculations are carried out by the Vienna ab initio simulation Package (VASP)^[1]^, with plane wave basis set with an energy cutoff of 500 eV, projector augmented wave (PAW) pseudopotentials^[2]^ and the generalized gradient approximation parameterized by Perdew, Burke, and Ernzerh of (GGA-PBE) for exchange-correlation functional^[3]^. Grimme’s semiempirical DFT-D3 scheme is employed to account for the dispersion correction^[4]^. The Brillouin zones were sampled by 4×4×1 k-point mesh. The model structures are fully optimized for the ionic and electronic degrees of freedom using the convergence criteria of 10^-4^ eV for electronic energy and 10^-2^ eV/Å for the forces on each atom.

**
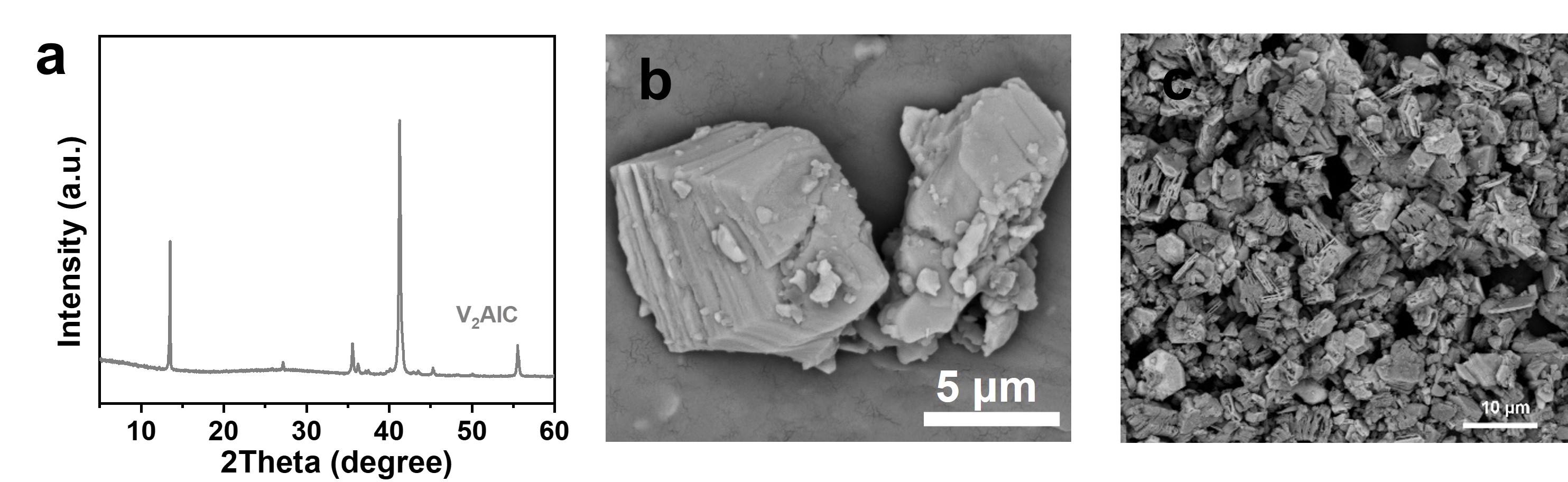
**

**Figure** S1. (a) XRD pattern of V_2_AlC. SEM images of (b) V_2_AlC, (c) V_2_CT_x_.

**
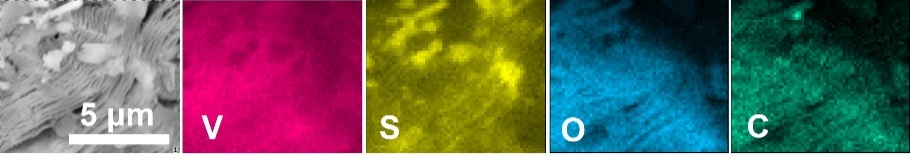
**

**Figure** S2. SEM image and EDS mappings of VS_2_/V_2_CT_x_.

**
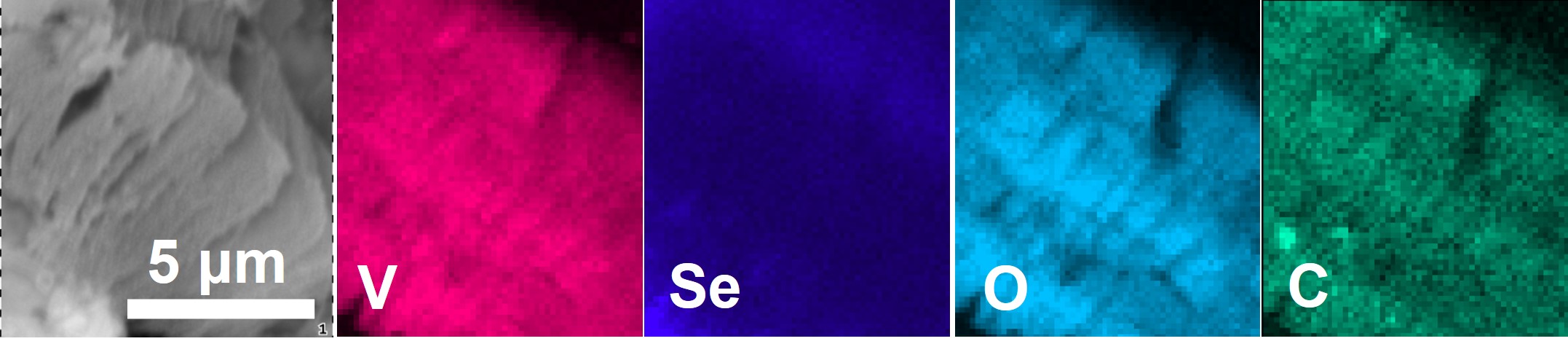
**

**Figure** S3. SEM image and EDS mappings of VSe_2_/V_2_CT_x_.


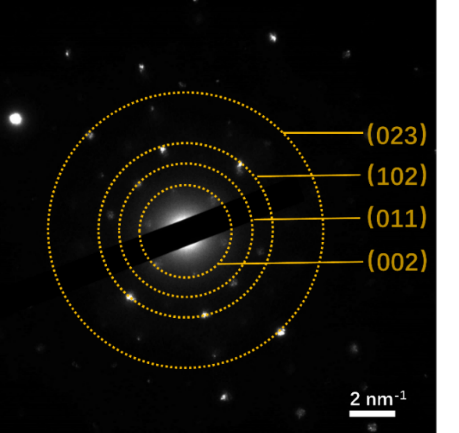


**Figure** S4. SAED image of VSSe/V_2_CT_x_.


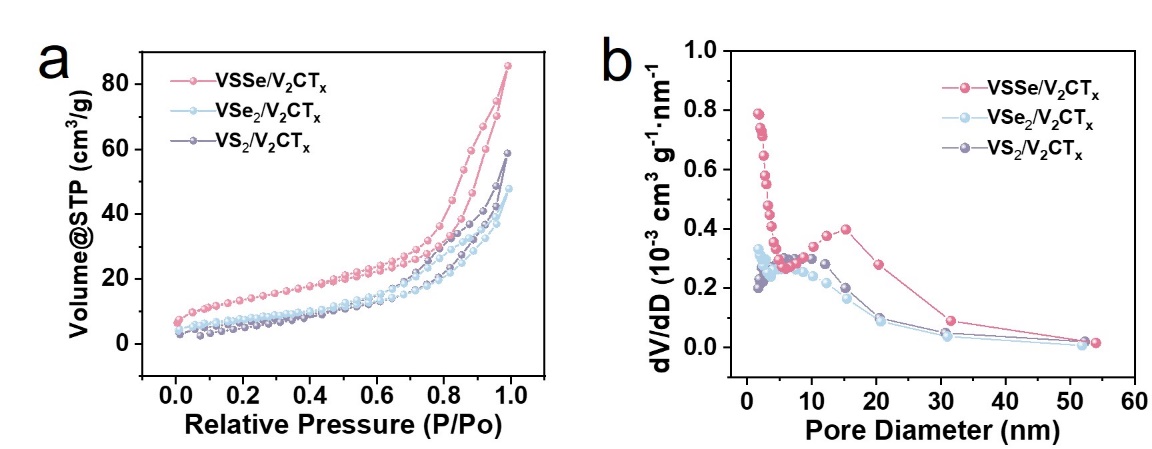


**Figure** S5. a) Nitrogen adsorption/desorption test of VSSe/V_2_CT_x_, VS_2_/V_2_CT_x_ and VSe_2_/V_2_CT_x_. and b) corresponding pore size distribution.


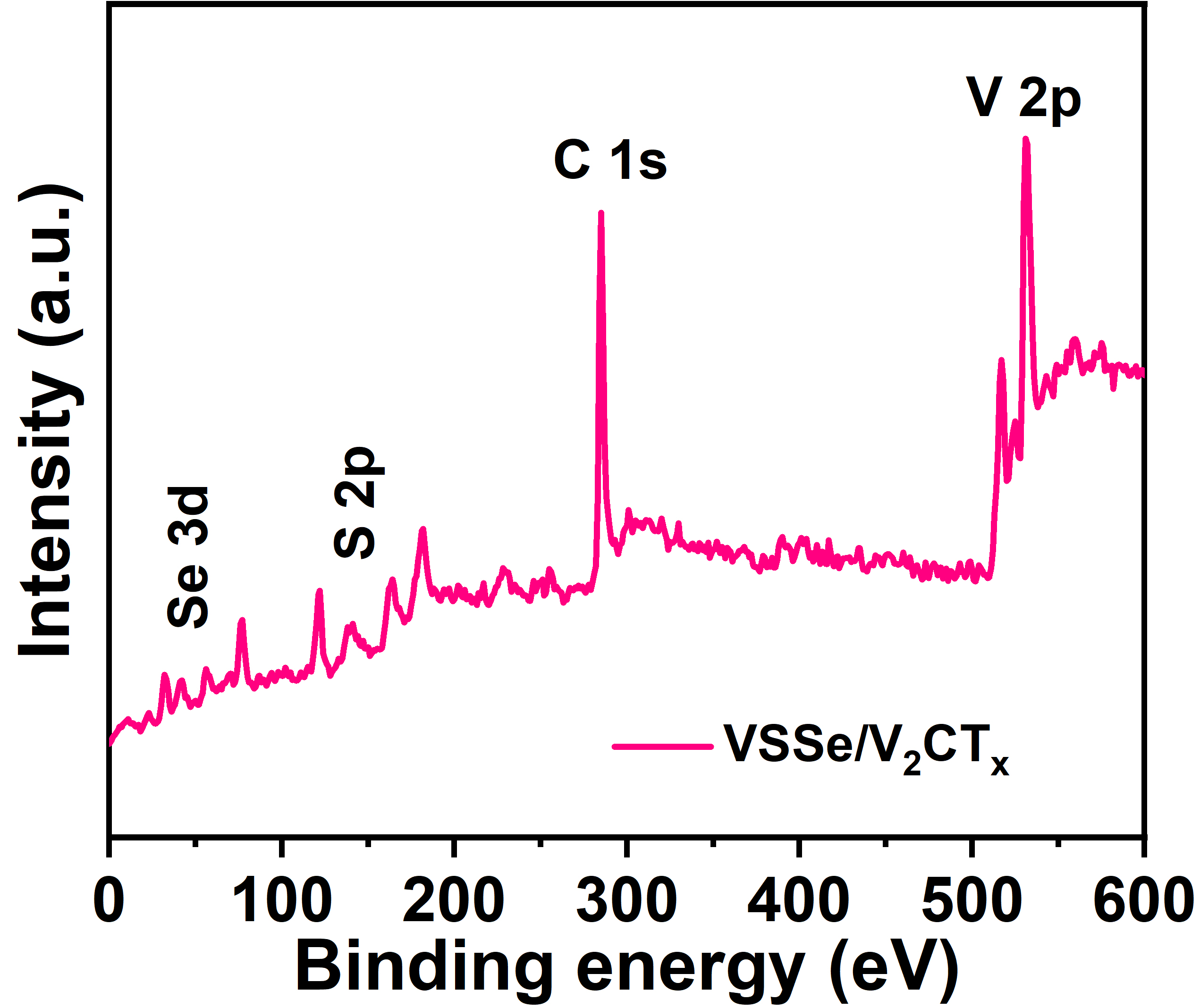


**Figure** S6. XPS survey spectrum of VSSe/V_2_CT_x_.


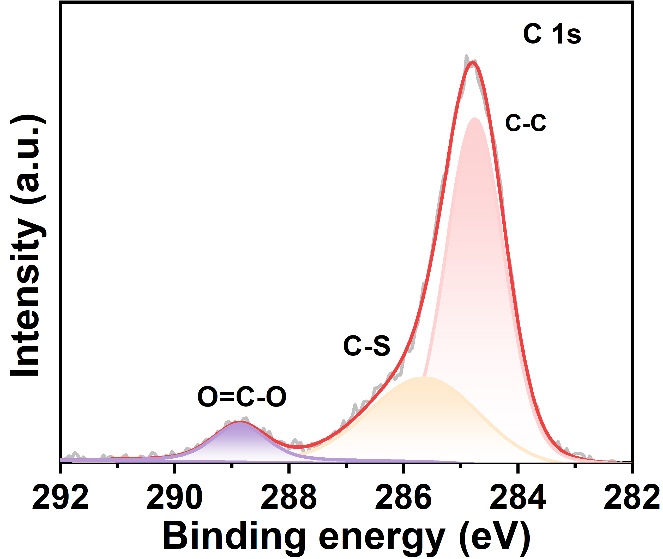


**Figure** S7. C 1s XPS spectrum of VSSe/V_2_CT_x_.


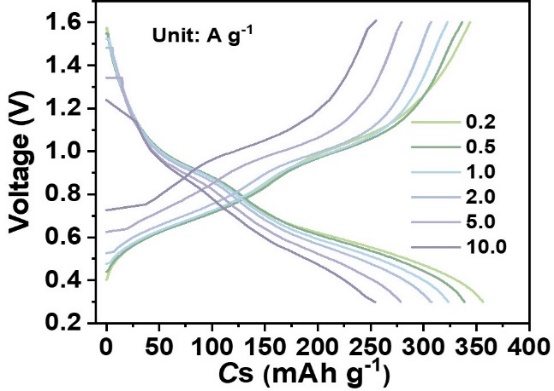


**Figure** S8. Discharge and charge curves of VSSe/V_2_CT_x_ at different current densities.


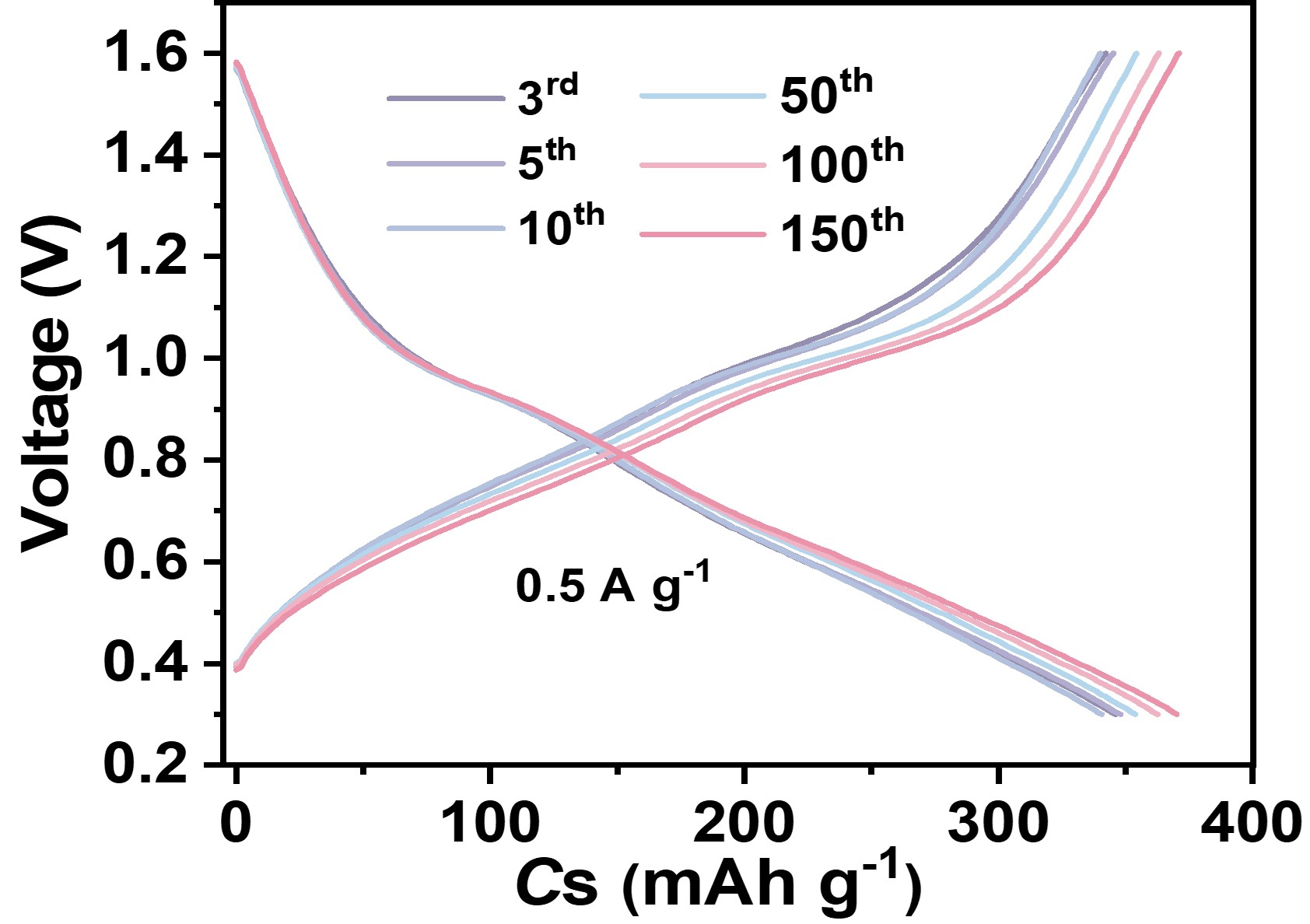


**Figure** S9. Corresponding charge/discharge curves with representative cycles.

**
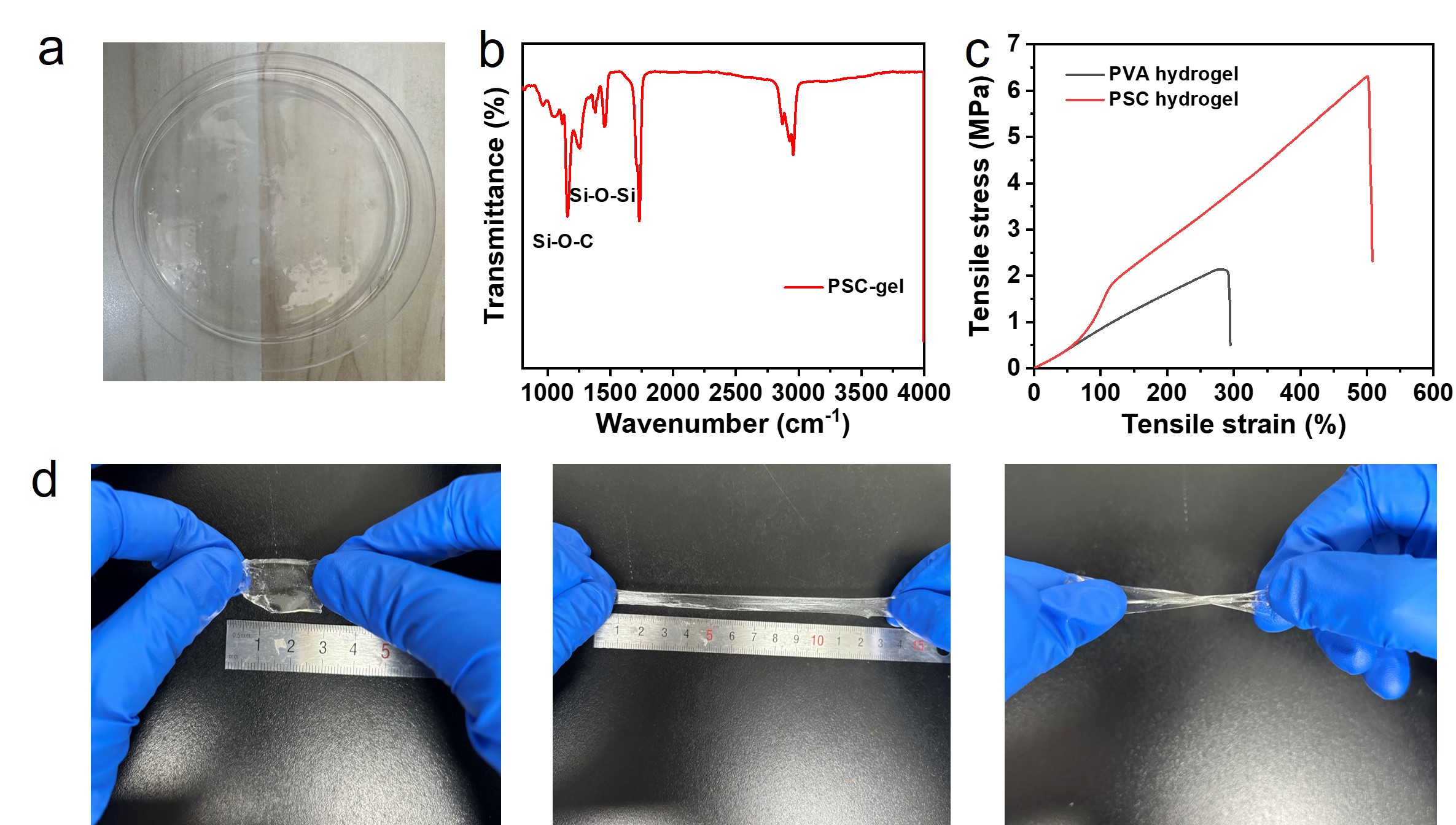
**

**Figure** S10. a) Digital photograph of PSC hydrogel. b) FTIR spectrum of PCS-gel. c) Stress-strain curves of PSC and PVA hydrogel. d) Digital photographs of PSC hydrogel at different stretching and twisting states.


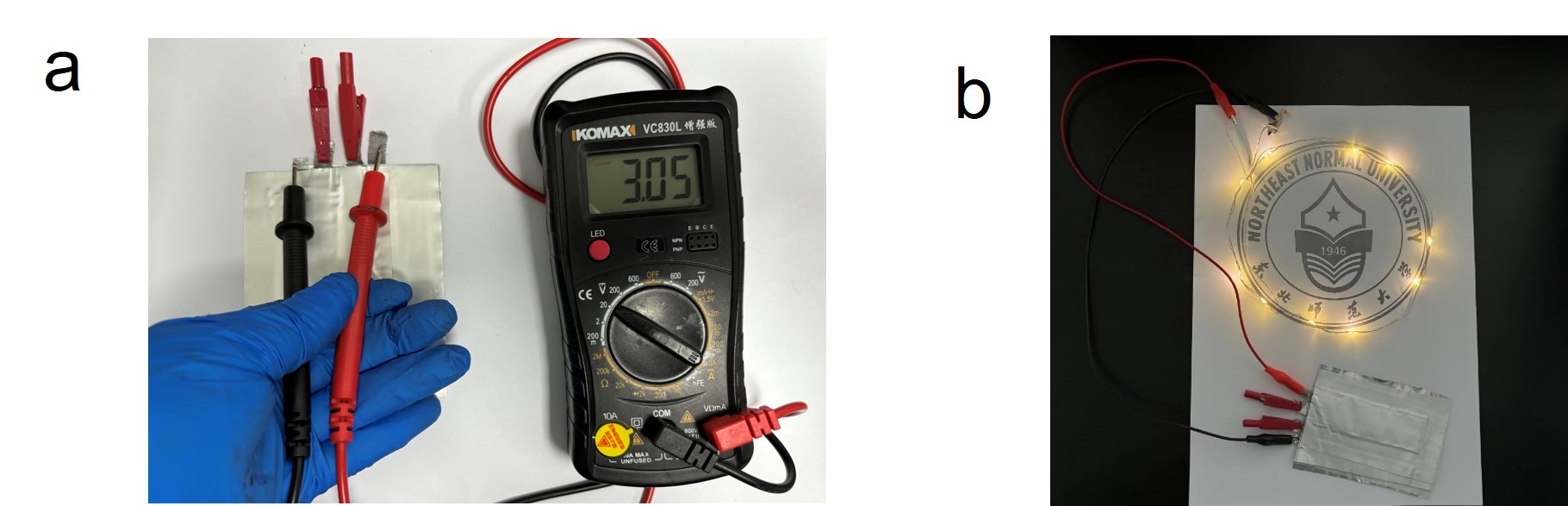


**Figure** S11. Digital photographs of a) the open circuit voltage and b) powering LEDs of two ZIBs connected in series.


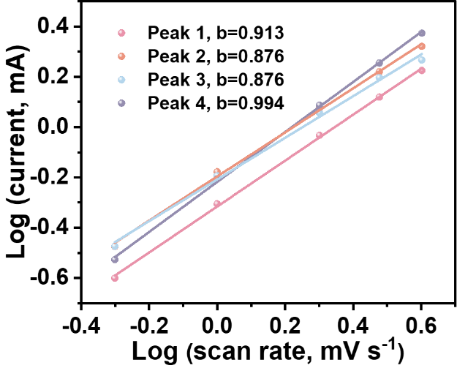


**Figure** S12. b values of the four peaks currents according to the CV curves at different scan rates.


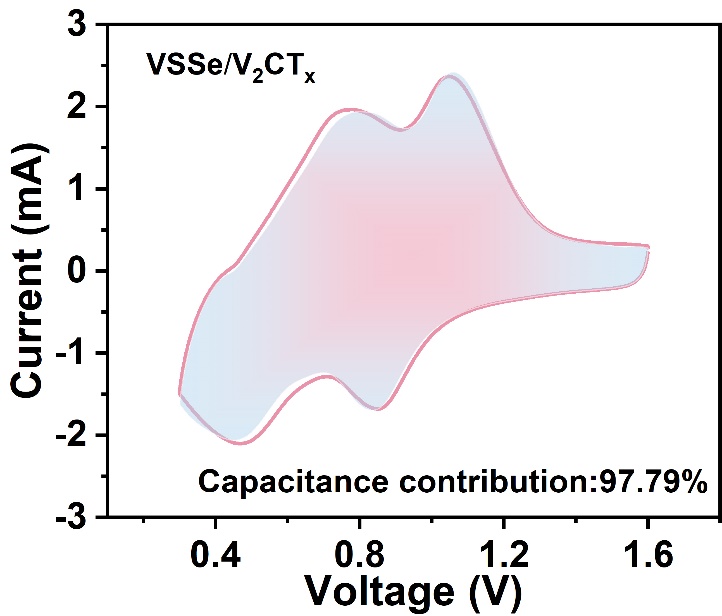


**Figure** S13. The typical CV profile with the pseudocapacitance ratio at 4 mV s^-1^.


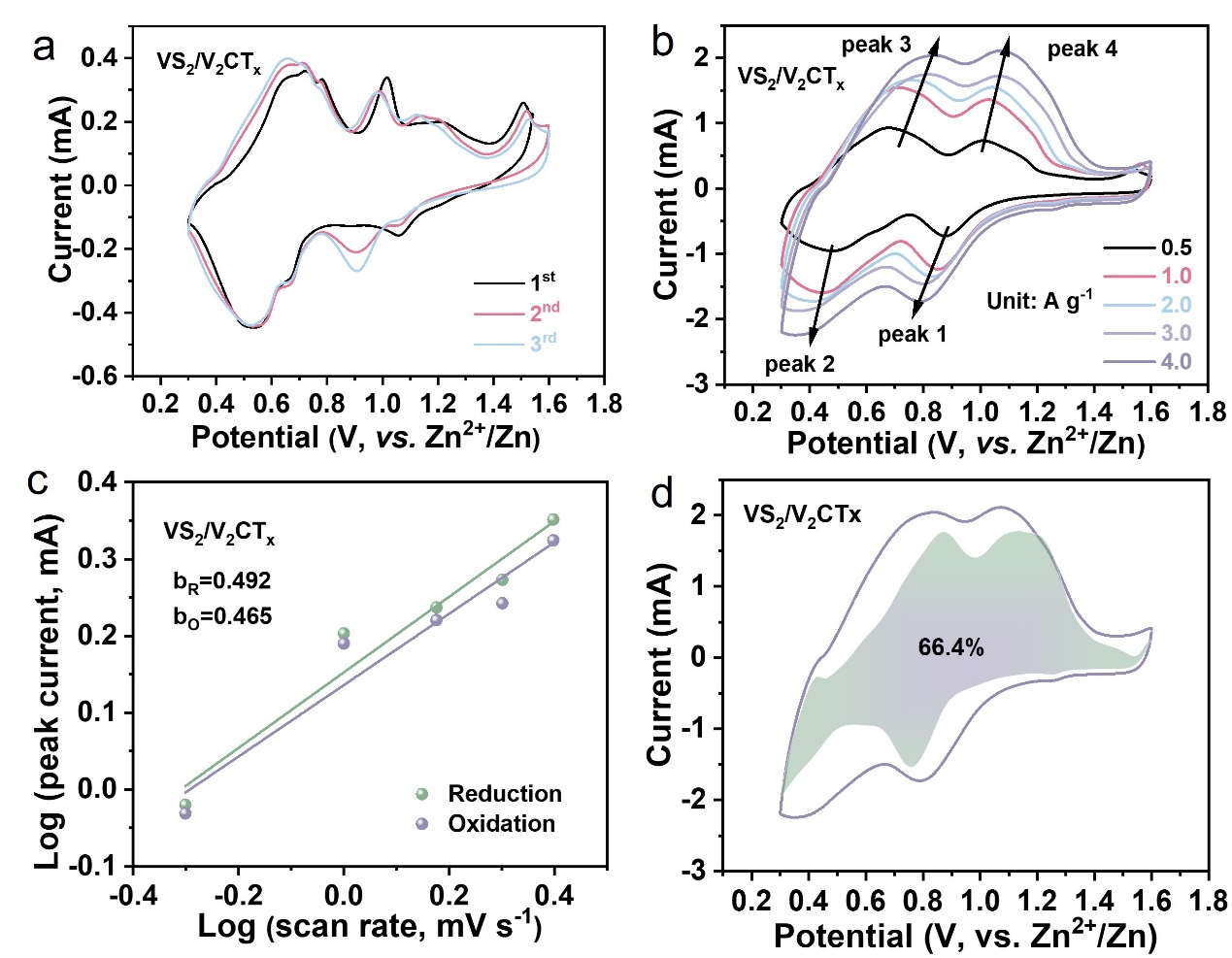


**Figure** S14. a) CV curves at 0.1 mV s^-1^ of VS_2_/V_2_CT_x_. b) CV profiles at gradient sweep rates from 0.5 to 4.0 mV s^-1^. c) b values of the four peaks currents according to the CV curves at different scan rates. d) The typical CV profile with the pseudocapacitance ratio at 4 mV s^-1^.


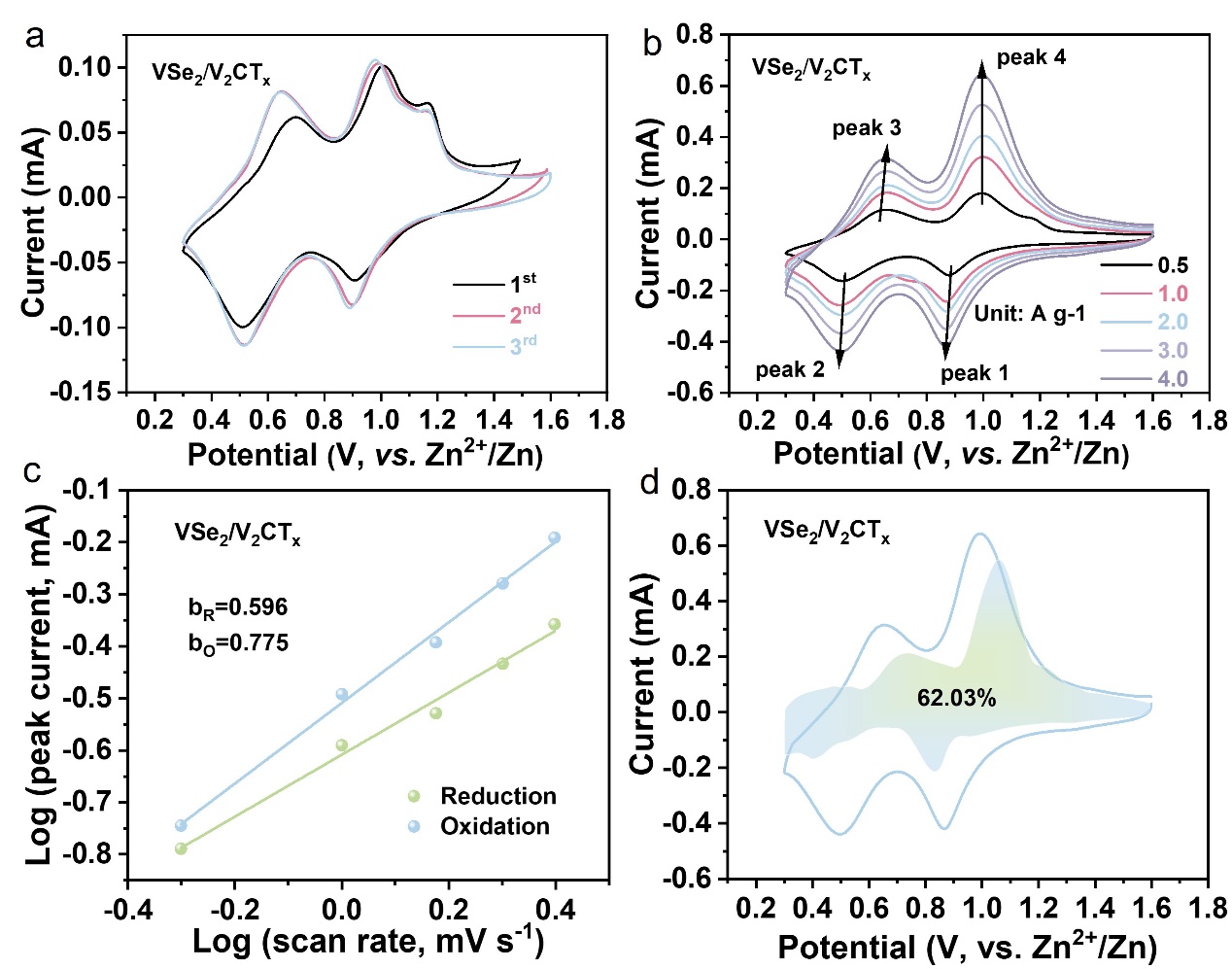


**Figure** S15. a) CV curves at 0.1 mV s^-1^ of VSe_2_/V_2_CT_x_. b) CV profiles at gradient sweep rates from 0.5 to 4.0 mV s^-1^. c) b values of the four peaks currents according to the CV curves at different scan rates. d) The typical CV profile with the pseudocapacitance ratio at 4 mV s^-1^.


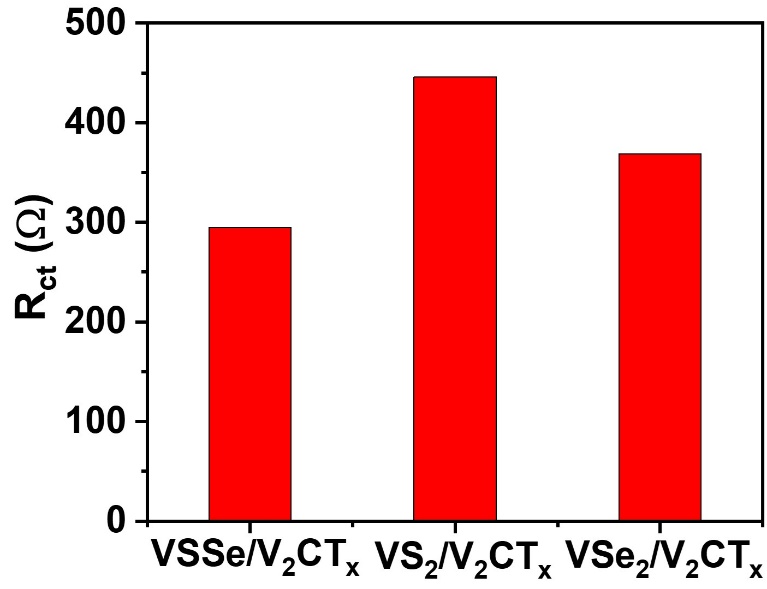


**Figure** S16. R_ct_ values of VSe_2_/V_2_CT_x_, VSe_2_/V_2_CT_x_, and VSe_2_/V_2_CT_x_.


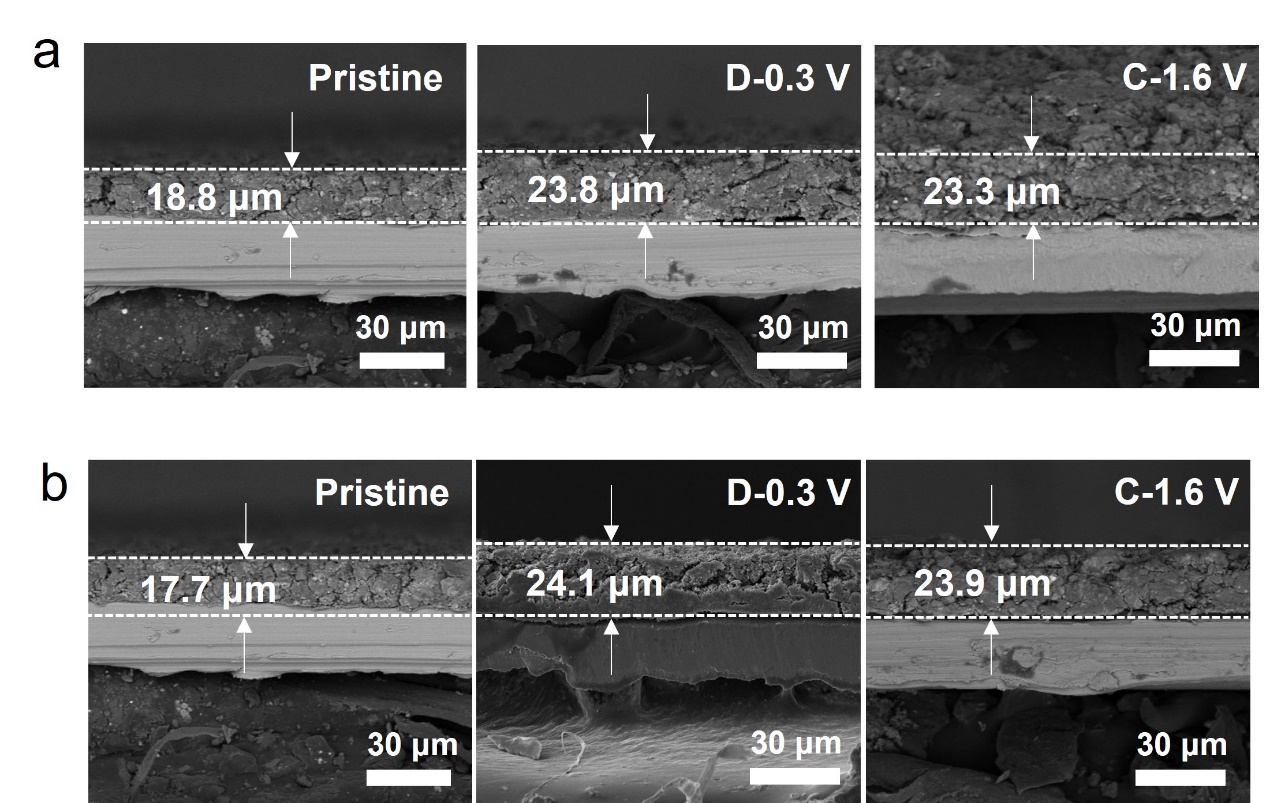


**Figure** S17. Cross-sectional SEM images of a) VSe_2_/V_2_CT_x_ and b) VS_2_/V_2_CT_x_ electrode at full discharge and charge states at 1 A g^-1^ after 100 cycles.


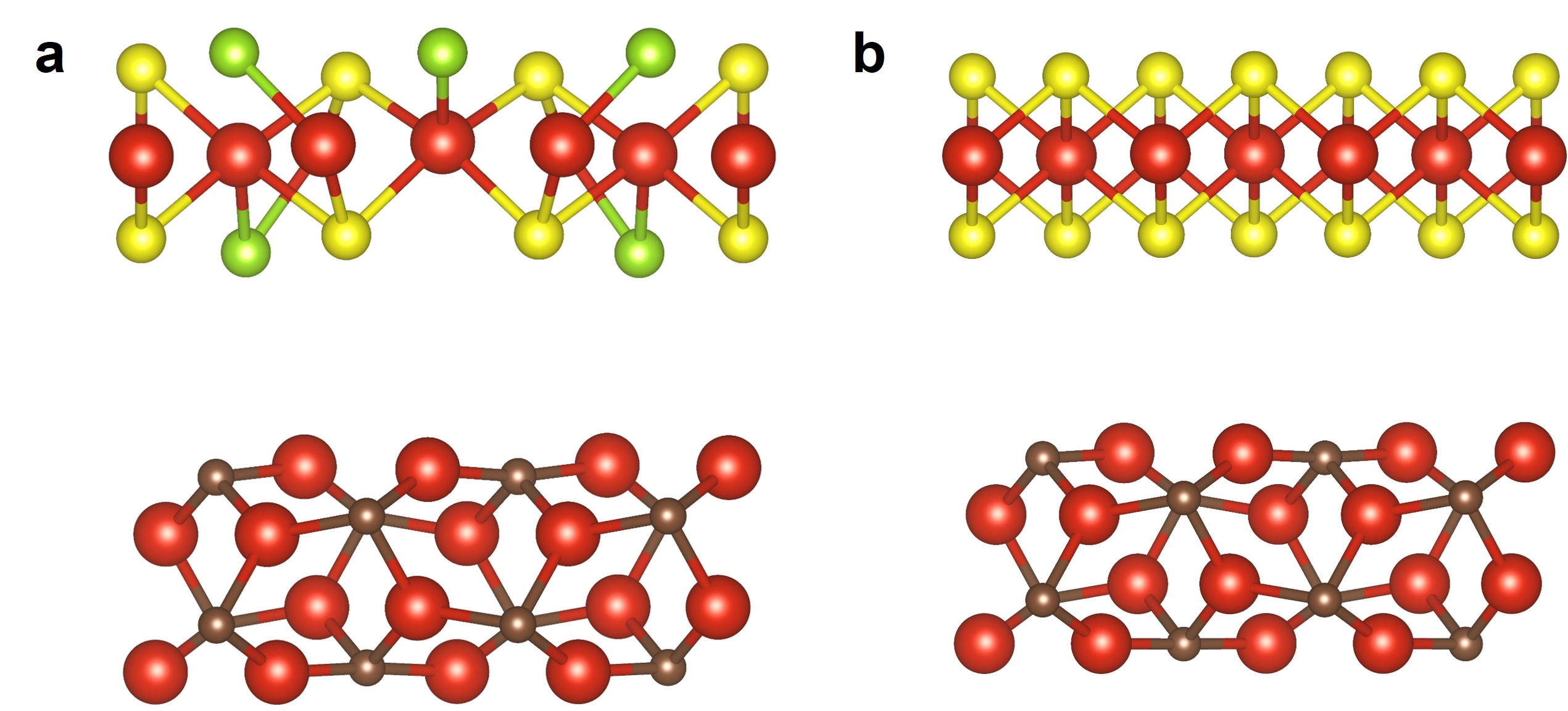


**Figure** S18. Optimized structure models of a) VSSe/V_2_CT_x_ and b) VS_2_/V_2_CT_x_.


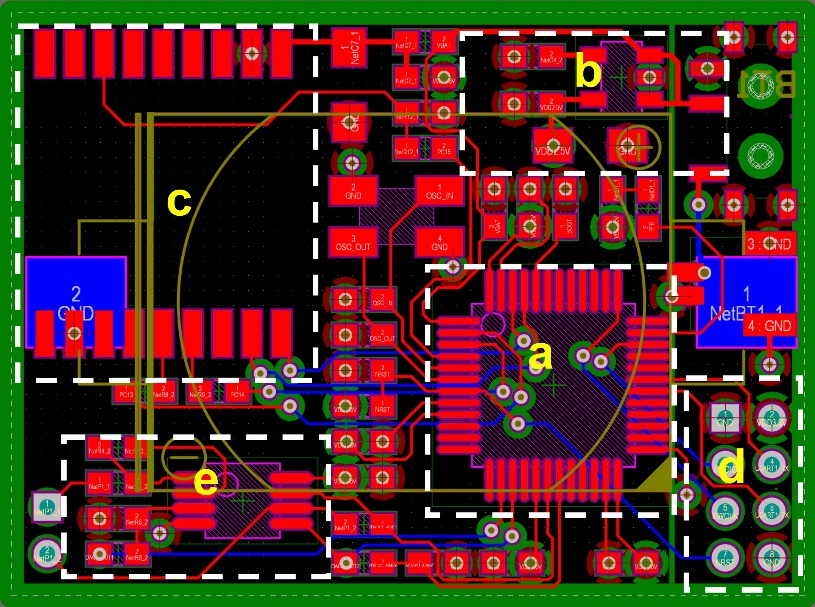


**Figure** S19. Image of PCB layout design. (a) STM32F373 Microcontroller unit. (b)Power management circuit. (c) Bluetooth module. (d) Program debugging interface.


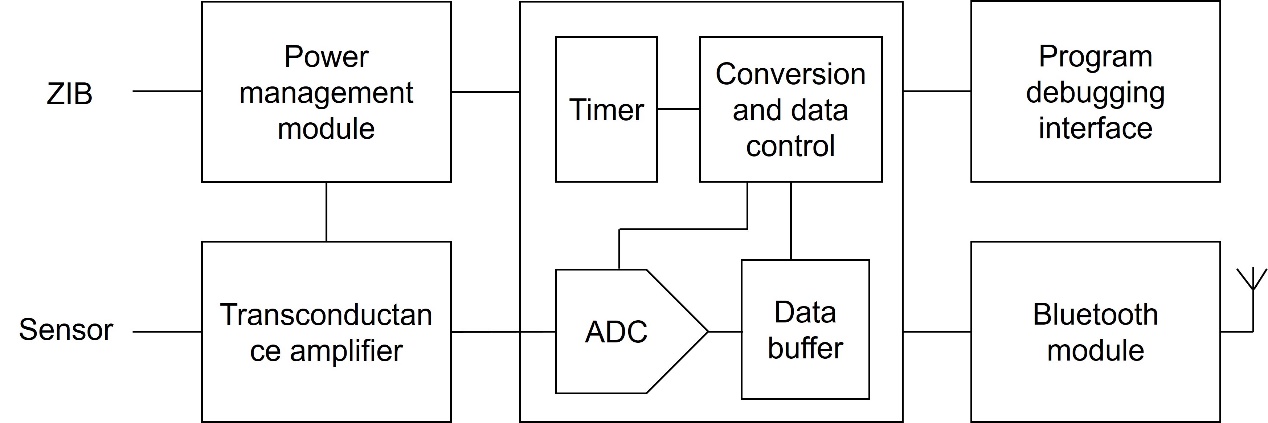


**Figure** S20. Schematic diagram of signal-conditioning circuit.


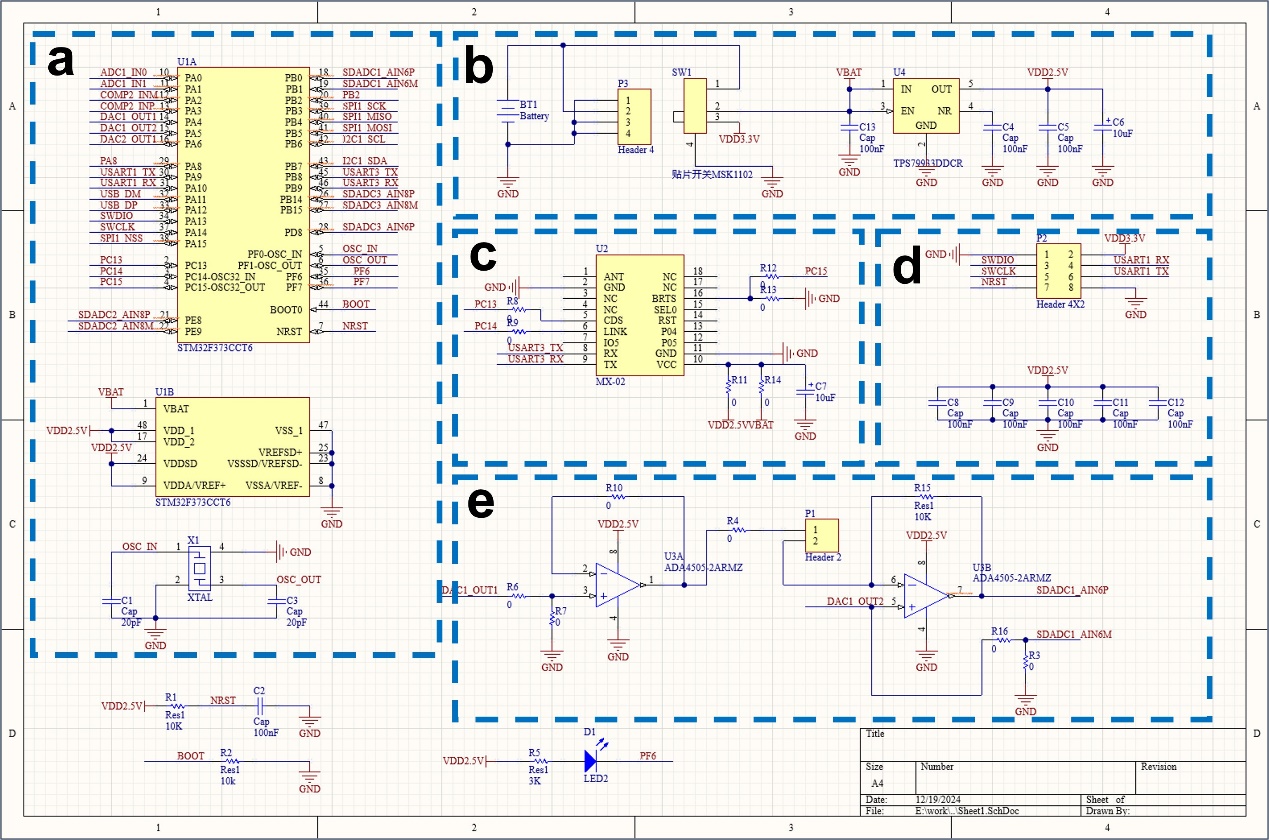


**Figure** S21. Image of PCB layout design. (a) STM32F373 Microcontroller unit. (b) Power management circuit. (c) Bluetooth module. (d) Program debugging interface. (e) Transconductance amplifier.


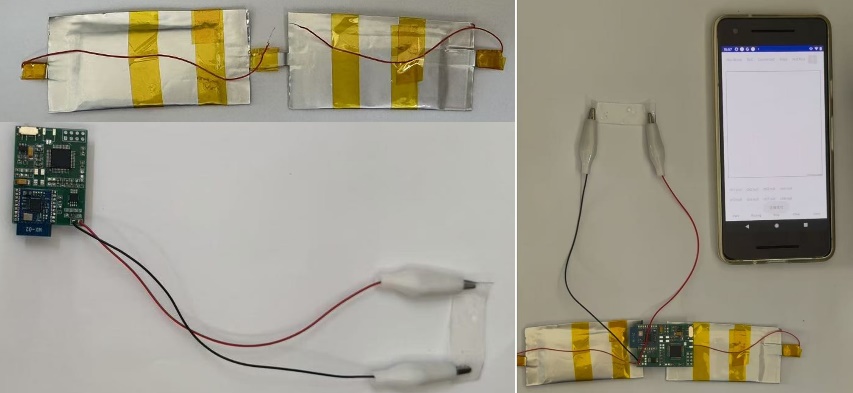


**Figure** S22. Digital photographs showcasing the integration process of the self-powered wearable sensing system.


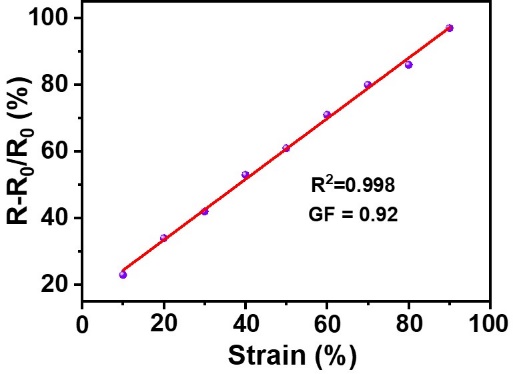


**Figure** S23. The relative resistance changes of the PSC hydrogel sensor as a function of the applied tensile strain (10-90%).

**Movie S1.** Demonstration of the self-powered strain sensor at different stretching strains with a stable signal output.

**References**

[1] J. F. G. Kresse, *Phys. Rev. B*. **1996,** *54*, 11169.

[2] D. J. G. Kresse, *Phys. Rev. B*. **1999,** *59*, 1758.

[3] K. B. John, P. Perdew, M. Ernzerhof, *Phys. Rev. Lett*. **1996,** *77*, 3865.

[4] S. Grimme, J. Antony, S. Ehrlich, and H. Krieg, *J. Chem. Phys*. **2010,** *132*, 154104.
